# Effective population size in a partially clonal plant is not predicted by the number of genetic individuals

**DOI:** 10.1111/eva.13535

**Published:** 2023-02-21

**Authors:** Roberta Gargiulo, Robin S. Waples, Adri K. Grow, Richard P. Shefferson, Juan Viruel, Michael F. Fay, Tiiu Kull

**Affiliations:** ^1^ Royal Botanic Gardens, Kew Richmond UK; ^2^ NOAA Fisheries, Northwest Fisheries Science Center Seattle Washington USA; ^3^ University of Washington Seattle Washington USA; ^4^ Department of Biological Sciences Smith College Northampton Massachusetts USA; ^5^ Organization for Programs in Environmental Sciences University of Tokyo Tokyo Japan; ^6^ School of Biological Sciences University of Western Australia Crawley Western Australia Australia; ^7^ Estonian University of Life Sciences Tartu Estonia

**Keywords:** conservation genetics, *Cypripedium calceolus*, double‐digest RAD sequencing, effective population size, microsatellites, partially clonal plants

## Abstract

Estimating effective population size (*N*_e_) is important for theoretical and practical applications in evolutionary biology and conservation. Nevertheless, estimates of *N*_e_ in organisms with complex life‐history traits remain scarce because of the challenges associated with estimation methods. Partially clonal plants capable of both vegetative (clonal) growth and sexual reproduction are a common group of organisms for which the discrepancy between the apparent number of individuals (ramets) and the number of genetic individuals (genets) can be striking, and it is unclear how this discrepancy relates to *N*_e_. In this study, we analysed two populations of the orchid *Cypripedium calceolus* to understand how the rate of clonal versus sexual reproduction affected *N*_e_. We genotyped >1000 ramets at microsatellite and SNP loci, and estimated contemporary *N*_e_ with the linkage disequilibrium method, starting from the theoretical expectation that variance in reproductive success among individuals caused by clonal reproduction and by constraints on sexual reproduction would lower *N*_e_. We considered factors potentially affecting our estimates, including different marker types and sampling strategies, and the influence of pseudoreplication in genomic data sets on *N*_e_ confidence intervals. The magnitude of *N*_e_/*N*_ramets_ and *N*_e_/*N*_genets_ ratios we provide may be used as reference points for other species with similar life‐history traits. Our findings demonstrate that *N*_e_ in partially clonal plants cannot be predicted based on the number of genets generated by sexual reproduction, because demographic changes over time can strongly influence *N*_e_. This is especially relevant in species of conservation concern in which population declines may not be detected by only ascertaining the number of genets.

## 1 INTRODUCTION

Monitoring effective population size (*N*_e_) is a powerful strategy for predicting and preventing the loss of genetic diversity in natural populations (Frankham, 1995; Jamieson & Allendorf, 2012). *N*_e_ is a pivotal parameter in evolutionary biology and conservation biology because of its direct relationship with genetic drift and its effects on other evolutionary forces, including selection and gene flow (Allendorf et al., 2013; Charlesworth, 2009; Crow & Kimura, 1970; Frankham et al., 2010; Wright, 1931). At the same time, *N*_e_ is one of the most difficult parameters to estimate directly from demographic data (Caballero, 1994; Campbell & Husband, 2005; Nunney & Elam, 1994; Orive, 1993; Yonezawa et al., 2004), and thus researchers have commonly preferred to estimate it using genetic methods (Gilbert & Whitlock, 2015; Luikart et al., 2010; Nadachowska‐Brzyska et al., 2021; Palstra & Ruzzante, 2008; Wang, 2005; Wang et al., 2016; Waples, 1989, 2005).

Among the factors that complicate the estimation of *N*_e_ are gene flow, nonrandom reproductive success among individuals, and overlapping generations (e.g., Hill, 1972; Jorde & Ryman, 1995; Neel et al., 2013; Nunney, 1993; Trask et al., 2017; Wang & Whitlock, 2003; Waples, 2002, 2016a, 2022; Waples & England, 2011). The research focused on evaluating biases in *N*_e_ estimates has been particularly active and has produced crucial insights for conservation applications (Luikart et al., 2010, 2021; Marandel et al., 2020; Palstra & Fraser, 2012; Ryman et al., 2019; Santiago et al., 2020; Wang et al., 2016; Waples, 2016b; Waples et al., 2014; Waples & England, 2011). Given the global biodiversity crisis, there is an urgency to obtain estimates of *N*_e_ (*N̂*_e_) for conservation practice and policy (Frankham, 2021; Hoban et al., 2021; Laikre et al., 2021).

Partial clonality is a widespread reproductive feature across all eukaryotes (Halkett et al., 2005; Krueger‐Hadfield et al., 2021; Stoeckel, Arnaud‐Haond, & Krueger‐Hadfield, 2021; Vallejo‐Marín et al., 2010). Partially clonal plants, in particular, represent one common, but relatively understudied, group of organisms for which there is often a striking discrepancy between the observed number of plants and the number of genetic individuals in a population (Alberto et al., 2005; Arnaud‐Haond et al., 2020; Honnay & Jacquemyn, 2008; Lozada‐Gobilard et al., 2021; Tepedino, 2012; Widén et al., 1994). The observed number of plants in partially clonal plants reflects the contribution of both sexual reproduction, which generates genetic individuals or genets, and clonal reproduction, which generates ramets that are replicates of the same genet (as in the most common type of clonal or vegetative growth, occurring in 80% of angiosperms; Klimeš et al., 1997; Vallejo‐Marín et al., 2010). Just as *N*_e_ is imperfectly predicted by population census size (*N*_C_) (Frankham, 1995; Palstra & Ruzzante, 2008; Waples et al., 2016), the number of ramets is notoriously a poor surrogate for *N*_e_ in a partially clonal plant population (Chung et al., 2004; Tepedino, 2012), as well as a poor indicator of levels of genetic diversity (Mandel, 2010; Raabová et al., 2015). It is worth noting that the number of ramets also encompasses juveniles, and thus may not equal *N*_C_, because the latter is most commonly defined as the number of mature individuals (e.g., Frankham, 1995; Luikart et al., 2010; Nunney, 1991, 1995). The number of genets, which can only be ascertained through genetic analysis, is generally expected to be closer to *N*_e_ than *N*_C_, albeit not equivalent.

It has been suggested that the relationship between clonality and *N*_e_ is not straightforward because of confounding factors linked to other life‐history traits (Campbell & Husband, 2005), such as lifespan and generation time (Nunney, 1993; Yonezawa, 1997), rate of selfing, and especially the variance of clonal and sexual reproductive contributions of individuals (Orive, 1993; Yonezawa et al., 2004). However, clonal reproduction alone should not cause any significant change in *N*_e_, unless it occurs at extremely high rates and generates fixed heterozygosity (Balloux et al., 2003).

A review based on 63 iteroparous (i.e., capable of reproducing multiple times in a lifetime) species showed that only two traits, namely age at maturity and adult lifespan, explained half of the variance in *N*_e_/*N*_C_, demonstrating that the evolutionary implications of these two traits are consistent across taxa (Waples et al., 2013; Waples et al., 2016; see also Lee et al., 2011). Most of the investigations of *N*_e_ in partially clonal plants are based on demographic estimators of *N*_e_, whereas exhaustive empirical comparisons of the number of ramets, number of genets, and *N̂*_e_ obtained through genetic analyses are rare (Chung et al., 2004). In general, genetic estimates of *N*_e_ are expected to be lower than demographic estimates because they combine the influence of all demographic factors (Nunney & Elam, 1994; Palstra & Ruzzante, 2008) which are difficult to account for simultaneously.

The partially clonal orchid *Cypripedium calceolus* L. (lady's slipper orchid) is a good model system to investigate how *N*_e_ changes depending on the balance between clonal and sexual reproduction. Some populations of the species are characterized by a ratio of sexual reproduction to clonal reproduction equal to 1:200, mainly as a result of limitations in seed germination or the absence of pollinators (Devillers‐Terschuren, 1999; Kull, 1998, 1999; but see García et al., 2010). Moreover, seedling survival is generally low, as in other terrestrial orchid populations (Shefferson et al., 2020), with a probability of seeds reaching maturity estimated as 10^−7^ in Polish populations (Nicolé et al., 2005). Nevertheless, genetic analyses employing traditional molecular markers (allozymes, AFLPs, and microsatellites) have shown moderately high levels of genetic diversity even in small populations (*N*_C_ < 500), and this has been mainly attributed to vegetative growth, genet longevity (30–100 years; Kull, 1999; 110–350 years according to Nicolé et al., 2005; with an age at reproductive maturity: 6–10 years old; Kull, 1999), and mating system by outcrossing (Brzosko et al., 2002; Fay et al., 2009; Gargiulo, Adamo et al., 2021; Kull & Paaver, 1997; Minasiewicz et al., 2018).

Tremblay et al. (2005) suggested that *N*_e_/*N*_C_ in orchids is particularly low because of pollinator‐related limitations, and this is consistent with the observation that an increase in the variance in reproductive success decreases *N*_e_ (Frankham, 1995; Nunney, 1991, 1993; Waples, 2016a). All else (i.e., generation length and age at maturity) being equal within a single species, populations in which sexual reproduction is less limited by pollinators (i.e., populations with a higher rate of sexual reproduction) should have a higher *N*_e_/*N*_C_ ratio than populations with little sexual reproduction. Although effective population size should not be significantly affected by clonal reproduction unless sexual reproduction is very rare (Balloux et al., 2003), the vegetative spread will imply that larger individuals may sexually reproduce more, thus increasing variance in reproductive success and lowering *N*_e_.

In the present study, we asked how clonal versus sexual reproduction affected the effective population size of two populations of *C. calceolus* with different demographic histories. We start from the theoretical expectation that clonal reproduction lowers *N*_e_ by increasing variance in reproductive success among individuals, and the constraints on sexual reproduction also lower *N*_e_ by causing only a few plants to reproduce. In two populations of the same species, we expect that when sexual reproduction is less constrained, *N*_e_/*N*_C_ would be larger. We used an exhaustive sampling strategy and analysed microsatellites and SNPs derived from double‐digest restriction site‐associated DNA sequencing (ddRADseq) to compare different sets of genetic estimates (which are influenced by different mutation rates and errors associated with different molecular marker types). We first assessed whether genetic data support the observation of different rates of clonal and sexual reproduction in the two populations. We then estimated contemporary *N*_e_ with the linkage disequilibrium method (Hill, 1981; Waples & Do, 2008) using both microsatellites and ddRADseq‐SNPs. We improved the precision of our *N̂*_e_ confidence interval by subsampling the number of loci, and we corrected the bias in *N̂*_e_ point estimates due to physical linkage among loci following Waples et al. (2016).

## 2 MATERIALS AND METHODS

### 2.1 Population sampling

The two Estonian populations of *C. calceolus* selected for the present study have been monitored annually since 1978 and 1985, respectively (Hurskainen et al., 2017; Kull, 1995, 1998, 2003). The continental population, hereafter “Ussisoo,” is characterized by a generally stable demography (Table S1) and little fruit set. The insular population, hereafter “Kõrgessaare,” occurs in a coastal forest on the Baltic Island of Hiiumaa at the border of a lagoon system (1–2 m above sea level) and includes abundant seedlings. Kõrgessaare is thought to have originated more recently than Ussisoo, probably around 100 years ago or less, after changes in the habitat type (Gargiulo et al., 2018; Kull & Paaver, 1997), with substantial population growth in the last few decades (Table S1).

**FIGURE 1:**
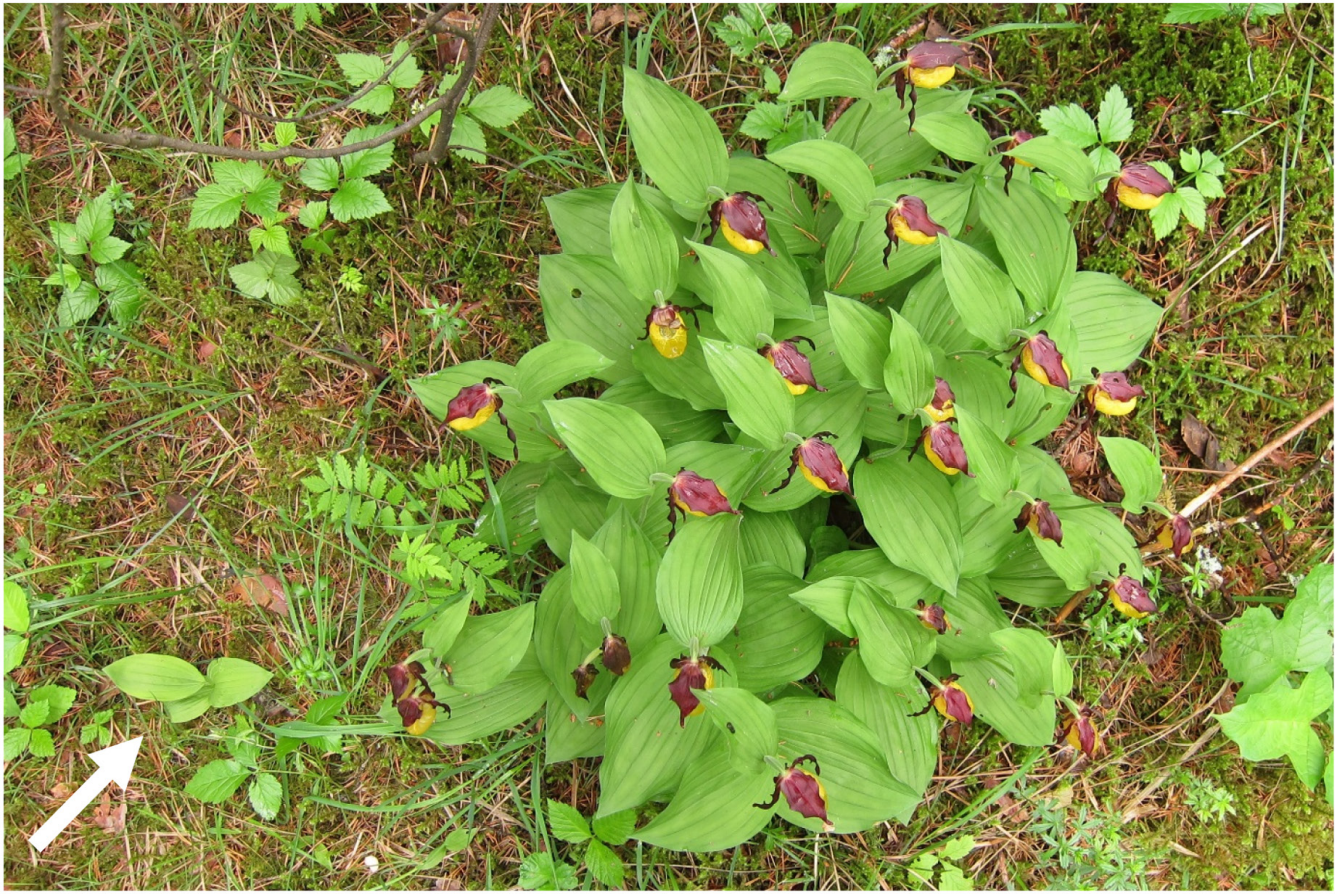
A clump of *Cypripedium calceolus* in Kõrgessaare. Note the juvenile (different genetic individual, or genet) in the bottom‐left corner.

Clonal growth in *C. calceolus* follows a phalanx strategy, with all ramets from the same clone (i.e., a clump) close to each other in a rounded shape (Figure 1). However, especially when understory vegetation is abundant, different clones may be difficult to distinguish; recruitment within a clump may also occur (Nicolé et al., 2005). All emerging ramets from every single clump were sampled for leaf tissue (nondestructive sampling) and stored in silica gel (Chase & Hills, 1991). In Ussisoo, we collected 451 ramets from 35 putative clumps (exhaustive sampling of all visible plants in the population), and in Kõrgessaare, we collected ~700 ramets from >40 putative clumps (exhaustive sampling of all visible plants at the random coordinates chosen). Sampling was carried out in June 2019, and the Ussisoo population was translocated in August 2019 due to the expansion of the adjacent road.

### 2.2 Microsatellite genotyping and analyses of multilocus genotypes

Genomic DNA was extracted with a modified CTAB method (Doyle & Doyle, 1987) and purified with a QIAquick PCR purification kit (QIAGEN, Manchester, UK). All samples were genotyped for 11 nuclear microsatellite (or simple sequence repeat, SSR) loci (Gargiulo et al., 2018; Minasiewicz & Znaniecka, 2014) following the protocol in Gargiulo et al. (2019) for amplification, allele calling and evaluation of scoring errors/null alleles. Samples belonging to the same populations/clumps were randomized at different steps of the analysis (DNA extraction, polymerase chain reaction, and capillary electrophoresis) to avoid batch effects (Bonin et al., 2004; Meirmans, 2015). The total SSR data set obtained (i.e., including all ramets) is hereafter indicated as “raw data set.” Multilocus genotypes (MLGs) were analysed in the *R* v4.0.5 (R Core Team, 2020) packages poppr v2.8.6 (Kamvar et al., 2014, 2015) and adegenet v2.1.3 (Jombart, 2008; Jombart & Ahmed, 2011) to obtain indices of genotypic diversity. In poppr, we identified identical MLGs and kept one representative for each MLG to generate the “MLG‐based clone‐corrected data set.”

Before proceeding with further analyses, we performed some checks aimed to avoid the overestimation of either clonal or sexual reproduction in the two populations. In poppr, we assessed (1) whether all replicates of the same MLG (i.e., putative clones) were truly part of the same genet and not randomly generated by sexual reproduction (*psex* probability; Parks & Werth, 1993; Arnaud‐Haond et al., 2007; Figure S2) and (2) whether each distinct MLG actually belonged to a distinct genet and was not an artefact deriving from scoring errors (Arnaud‐Haond et al., 2007; Halkett et al., 2005). To assess the second point, we estimated a genetic distance threshold for collapsing MLGs potentially deriving from scoring errors, using the function *cutoff_predictor* based on Bruvo's distances (see details in Figure S3). After establishing the genetic threshold, we recomputed indices of genotypic diversity in the data set obtained by collapsing potentially identical MLGs in multilocus lineages (MLLs; the related data set is hereafter indicated as “MLL‐based clone‐corrected data set”) and produced a minimum spanning network to visualize relationships among MLLs.

In the package RClone v1.0.2 (Bailleul et al., 2016), we computed Pareto (power law) distributions (β) for the two populations. The Pareto distribution is used to describe the distribution of ramets into MLLs and is influenced by both genotypic (clonal) richness and evenness. For example, when MLLs have comparable sizes (high evenness), the Pareto plot will result in a steeper slope (high β) (see Arnaud‐Haond et al., 2007; Stoeckel, Porro, & Arnaud‐Haond, 2021).

We used exact tests in Genepop v4.5.1 (Raymond & Rousset, 1995; Rousset, 2008) with default parameters to assess deviations from Hardy–Weinberg proportions (potentially indicative of deviations from random mating), and we compared the departures towards heterozygosity excess and deficit and calculated locus‐level *F*_IS_. The number of private alleles, observed heterozygosity (*H*_O_) and unbiased expected heterozygosity (u*H*_E_), were computed in GeneAlEx v6.5 (Peakall & Smouse, 2006, 2012), and private allelic richness was computed in HP‐Rare (Kalinowski, 2004, 2005) on the MLG‐based and the MLL‐based clone‐corrected data sets. To test the hypothesis that outbred (i.e., highly heterozygous) plants have higher fitness (Alberto et al., 2005; Hämmerli & Reusch, 2003; but see Shefferson et al., 2018), we evaluated the correlation between individual heterozygosity and the number of ramets representing each MLL, as we can assume that plants with more ramets have lived for a long time. Individual heterozygosity was computed as the proportion of typed loci for which an individual clone was heterozygous (Hämmerli & Reusch, 2003). The allelic richness and *F*_IS_ values per population were computed in FSTAT v.2.9.3 (Goudet, 2001). To assess the occurrence of nonrandom associations among loci, we computed the index of association (r_d_) in poppr.

To check for the occurrence of recent migrants and internal population structure, which can bias *N̂*_e_, we analysed the “MLG‐based clone‐corrected data set” through Bayesian clustering in Structure v.2.3.4 (Pritchard et al., 2000) using the Admixture model and no prior on sampling sites. We ran the analysis with 10^5^ burn‐in, 10^5^ MCMC replicates, and 20 iterations, and tested *K* values (number of genetic clusters) ranging from 1 to 5. We evaluated the most likely *K* using the LnPr(*X*|*K*) method (Pritchard & Wen, 2003) and the Δ*K* Evanno method (Evanno et al., 2005) in Structure Harvester (Earl & vonHoldt, 2012). The results were summarized in CLUMPAK (Kopelman et al., 2015).

### 2.3 Analysis of double‐digest RAD sequencing data

We used the double‐digest RAD sequencing (ddRADseq) protocol and data set obtained as in Gargiulo, Kull, et al. (2021). The data set included 31 ramets from Ussisoo, each collected from a different clump, and 32 ramets from Kõrgessaare (Table S2) all representing different clumps except three pairs of putative “biological replicates.” Each pair of biological replicates includes ramets at short distances that may belong to the same genet (pairs: EK308‐EK549, EK333‐EK336, and EK471‐EK206). We used one sample from Ussisoo as a technical replicate throughout ddRADseq library preparation and sequencing. De novo locus assembly was conducted in Stacks v2.4 (Catchen et al., 2013; Rochette et al., 2019) as detailed in Gargiulo, Kull, et al. (2021). We used the *populations* program of Stacks to filter ddRADseq data depending on the different assumptions and software programmes required in our downstream analyses, as detailed in Table S3, in addition to filtering mostly aimed at reducing the influence of repetitive and paralogous loci expected in the large genome of *C. calceolus* (Gargiulo, Kull, et al., 2021). We checked the occurrence of loci potentially under the effect of selection using BayeScan v2.1 (Foll et al., 2010; Foll & Gaggiotti, 2008), to exclude them from the subsequent analyses focused on neutral demographic processes. To avoid that the reduction of informative sites (due to our filtering strategies) determined the detection of false positives (Lotterhos & Whitlock, 2014), we performed the analysis on the “r80 data set” (i.e., the data set including loci shared by 80% of the samples in each population; see Table S3). Only the first SNP at each locus was included in the analysis (option in the Stacks *populations* program*: write‐single‐snp*). We set the prior odds of neutrality at 1000, the false discovery rate at 0.05, and the chain parameters at default values. Potential deviations from the Hardy–Weinberg proportions were evaluated in Stacks *populations* and in vcftools v0.1.16 (Danecek et al., 2011) using exact tests, after excluding *F*_ST_‐outlier loci (see also Table S3). P‐values for the multiple comparisons were corrected using the *p.adjust* function in R, using the false discovery rate method. After excluding the loci deviating from the Hardy–Weinberg proportions, we estimated the average nucleotide *diversity* (π), *H*_O_, *H*_E_, *F*_IS_, fixation index (*F*_ST_), and the number of private alleles in Stacks.

Fine‐scale genetic structure associated with the SNPs data set was evaluated in fineSTRUCTURE (Lawson et al., 2012) and visualized in RADpainter (Malinsky et al., 2018; i.e., fineRADstructure). The model implemented in fineRADstructure assumes linkage disequilibrium (LD) among SNPs, although a possible limitation of our data set is the relatively large size of our loci (>250 bp), so we cannot exclude historical recombination. Samples with high percentages of missing data were removed from the data set, as they may bias the estimation of coancestry coefficients (sensu Malinsky et al., 2018). We used the Rscript *reorderLD.R* to reorder loci based on their LD, sampling RAD‐tags variants 500 times, and then we ran fineSTRUCTURE with 100,000 burn‐in and 100,000 MCMC iterations, keeping every 1000th sample. Nearest neighbour haplotype relationships based on the coancestry values among pairs of samples were visualized in *R* using the script provided by Malinsky et al. (2018).

### 2.4 Estimation of effective population sizes *N*_e_

We employed the software NeEstimator v2.1 (Do et al., 2014) to estimate contemporary *N*_e_ using the linkage disequilibrium method (LD*N*_e_; Hill, 1981; Waples & Do, 2008). Confidence intervals for *N̂*_e_ were obtained by jackknifing over samples (Do et al., 2014; Jones et al., 2016; Waples et al., 2021). Both marker types (SSRs and SNPs) were analysed, on genet‐level data only, because linkage disequilibrium at the ramet‐level would be mainly affected by clonal reproduction and not by genetic drift. Below, we detail the analytical procedure for both marker types, including some caveats and the corrections aimed to improve both the precision and the accuracy on *N̂*_e_.

#### 2.4.1 SSRs

We analysed both the MLL‐based and the MLG‐based clone‐corrected data sets by excluding singletons (i.e., alleles only occurring in one heterozygote), and using different thresholds to screen out rare alleles (p‐crit for allele frequencies equal to 0.05, 0.02, 0.01, and 0) (Waples et al., 2016; Waples & Do, 2010). As we used an exhaustive sampling strategy (i.e., of all visible ramets/genets, except very young seedling stages and protocorm stages that are not visible overground), we assumed that the sampled cohorts approached the generation length of the species. Therefore, our *N̂*_e_ estimated from microsatellites should be close to the true *N*_e_, although a downward bias of at least 10% associated with mixed‐age adult sampling cannot be ruled out (Waples et al., 2014). Note that we consider the genet age in the case of a partially clonal plant such as *C. calceolus*. Estimates based on a single cohort have been shown to represent the effective number of breeders (*N*_b_) rather than *N*_e_ (Waples, 2005; Waples et al., 2014). Unfortunately, we could not obtain an estimate of *N*_b_ by analysing single cohorts as, even if the clump size (in a number of ramets) of *C. calceolus* can be predictive of the age of the clump, some clumps only produce one or two ramets over the years (T. Kull, pers. obs.). The occurrence of migration and population structure may be another source of bias in the estimation of *N*_e_ (Palstra & Ruzzante, 2008; Ryman et al., 2014, 2019). The LD method is robust to some population structure and to migration rates <0.1, whereas for higher migration rates, *N̂*_e_ approaches the *N*_e_ of the metapopulation (Waples & England, 2011; see also Gilbert & Whitlock, 2015). To evaluate the influence of potential migrants on *N̂*_e_, we removed the admixed individuals as detected in Structure for both *K* = 2 and *K* = 3 (displaying a proportion of admixture >20%, see Section 3) and recomputed *N̂*_e_.

#### 2.4.2 SNPs

The SNPs data set only included one SNP at each locus (option in the Stacks *populations* program*: write‐random‐snp*, see Table S3) to reduce the influence of physical linkage among sites. We used different p‐crit values for allele frequencies (i.e., 0.05, 0.02, 0.01, and 0) and excluded singletons. The LD*N*_e_ method implemented in NeEstimator is particularly robust with RADseq data when the number of samples is ≥30 (Nunziata & Weisrock, 2018). As nonrandom missingness due to allele dropout may bias the *N*_e_ estimation (Marandel et al., 2020), we previously evaluated the correlation between the proportion of missing data and *F*_IS_ at each locus, using Spearman's rank correlation test.

To compare confidence intervals for *N̂*_e_ generated by jackknifing over samples with parametric confidence intervals, which generally will be too narrow when thousands of loci are used (Waples, 2021; Waples et al., 2021), we subsampled the SNPs data set by generating 40 random whitelists of 800 loci in Stacks *populations* (with one SNP per locus, see Table S3) and analysed these subsets in NeEstimator. In addition, to further reduce potential biases due to the physical linkage among loci, we divided the point *N̂*_e_ by 0.098 + 0.219 × ln(10), as detailed in Waples et al. (2016), where 10 represents the haploid chromosome number of *C. calceolus*.

To account for the confounding effect of migrants and/or population structure, we removed a potentially admixed individual detected in fineRADstructure from the data set and recomputed *N̂*_e_.

## 3 RESULTS

### 3.1 Microsatellite genotyping and analyses of multilocus genotypes

The raw data set was composed of 1123 samples, including 451 samples from Ussisoo and 632 from Kõrgessaare, with a negligible percentage of missing data (Figure S1). When replicated MLGs were excluded, the MLG‐based clone‐corrected data set counted 66 MLGs in Ussisoo and 191 in Kõrgessaare (Table 1).

**TABLE 1:** Genotypic parameters associated with the SSR data set for the two populations of *Cypripedium calceolus* analysed in this study.

Population	*N* (raw data set)	#MLGs – #MLLs			eMLGs			G			E.5			Lambda	rbarD	Genotypic richness
		#MLGs	#MLLs‐threshold 0.011	#MLLs‐threshold 0.028	MLGs	MLLs‐threshold 0.011	MLLs‐threshold 0.028	MLGs	MLLs‐threshold 0.011	MLLs‐threshold 0.028	MLGs	MLLs‐threshold 0.011	MLLs‐threshold 0.028			
Ussisoo	491	66	66	59	66	66	59	29.3	29.3	28.3	0.76	0.76	0.78	0.97	0.11	0.13
Kõrgessaare	632	191	179	157	162	153	136	47.4	47.4	44.6	0.54	0.56	0.59	0.98	0.05	0.30
Total	1123	257	245	216	161	157	145	75.8	75.3	72.2	0.63	0.64	0.67	0.99	0.14	

Abbreviations: E.5, evenness; G, Stoddart and Taylor's Index of MLG diversity; Lambda, Lambda–Simpson's index; MLGs, multilocus genotypes; MLLs, multilocus lineages; eMLG, the number of expected MLG at the smallest sample size ≥10 based on rarefaction; rbarD, the standardized index of association.

*Note*: No identical multilocus genotypes were detected across populations.

**FIGURE 2:**
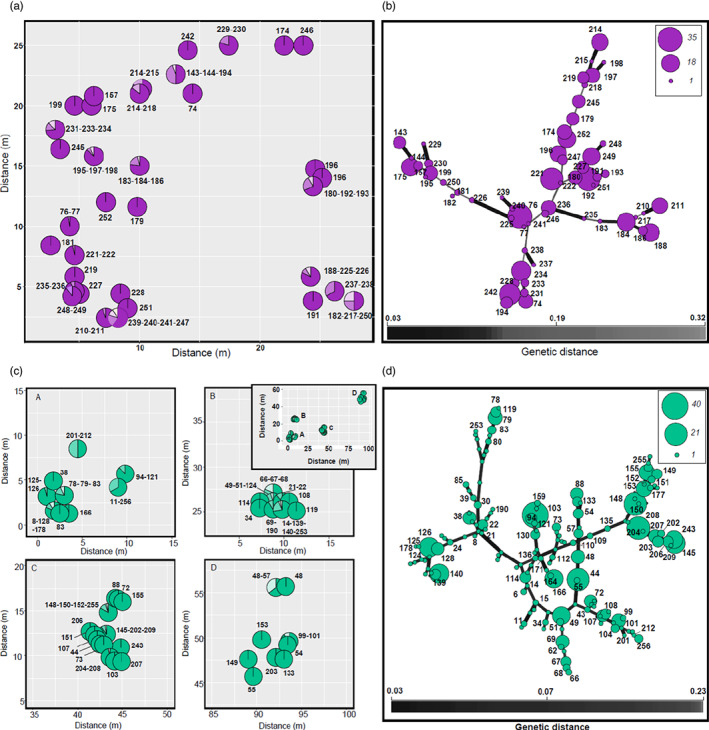
Maps representing the spatial distribution of the sampled clumps in Ussisoo (a) and Kõrgessaare (c); minimum spanning network (MSN) among multilocus lineages (MLLs) in Ussisoo (b) and Kõrgessaare (d). Each MLL is indicated by a code; pie sizes in the MSN are proportional to the number of ramets representing each MLL. Codes for the MLLs represented by isolated, single ramets are not shown for Kõrgessaare (and not reported in the spatial map). The inset in (c) refers to the entire sampling area in Kõrgessaare.

To avoid the overestimation of clones in the two populations, we evaluated whether all replicates of the same MLG (i.e., putative clones) were truly part of the same genet and not generated by chance due to sexual reproduction. The probability of encountering a genotype more than once by chance (single method) indicated that multiple MLGs are part of a single genet (*p*‐value associated with *p*sex ≪ 0.05), except in five cases in Kõrgessaare (Figure S2a). Results obtained by employing the multiple method corroborated the discrepancy between Ussisoo and Kõrgessaare, with single MLGs arising by sexual reproduction more frequently represented in the second population (Figure S2b). To avoid the overestimation of sexual reproduction, on the other hand, we evaluated the thresholds for collapsing MLGs potentially representing scoring errors, based on genetic distances. The threshold was ~0.011 when genotypes with missing data were included in the data set and 0.028 when genotypes with missing data were excluded (Figure S3). The number of MLLs obtained with this approach was 59 in Ussisoo and 157 in Kõrgessaare. Most of the clumps included ramets with identical MLGs (i.e., truly clonal ramets; Figure 2). In a few cases, for example, when boundaries among clumps were less clear, different MLLs deriving from sexual recruitment were found at small distances (i.e., <50 cm; Figure 2). Clump sizes were similar between the two populations, ranging from 1 to ~40 ramets. The Pareto β obtained in RClone (Figure S4) was low, reflecting the occurrence of clonal reproduction. Pareto plots showed a similar trend in both populations, reflecting lower genotypic richness/higher evenness in Ussisoo and higher genotypic richness/lower evenness in Kõrgessaare (Table 1).

After applying a Bonferroni correction for multiple testing, we found evidence for deviations from Hardy–Weinberg proportions and high variance in *F*_IS_, differentially occurring depending on the data set considered (i.e. raw data set, MLL‐based clone‐corrected data set, and MLG‐based clone‐corrected data set, see Table S4). When removing clones, most of these deviations disappeared. In particular, Ussisoo still exhibited an excess of heterozygotes at locus Ccal_25, whereas Kõrgessaare showed an excess of heterozygotes at locus IM30B and a deficit of heterozygotes at locus IK9. One heterozygote deficit was locus‐specific (Ccal_53) in both populations, possibly resulting from allele dropout. Twenty‐six private alleles were found in Ussisoo (rarefied private allelic richness: 1.86), in contrast with the four private alleles found in Kõrgessaare (rarefied private allelic richness: 0.51; Table 2); a comparison with the data set including other Eurasian populations (Gargiulo et al., 2019) showed that all private alleles found in Ussisoo occur elsewhere in Eurasia (Table S5; except the alleles at locus Ccal_50, for which large‐scale data are not available). In Kõrgessaare, allele 127 at Ccal_25 was found in one isolated ramet (single‐ramet plant). Observed and expected heterozygosities at the genet‐level were 0.56 and 0.59 in Ussisoo and 0.39 and 0.41 in Kõrgessaare (Table 2). There was no correlation between individual‐genet heterozygosity and the number of ramets representing that genet (*R* = 0.13, *p* = 0.34 at Ussisoo and *R* = 0.061, *p* = 0.45 in Kõrgessaare; Figure S5). Allelic richness based on the minimum sample size (64 diploid individuals) was 5.9 in Ussisoo and 3.4 in Kõrgessaare; *F*_IS_ was 0.04 and 0.06 when considering, respectively, the MLG‐based and the MLL‐based clone‐corrected data sets, in both populations (Table 2). The r_d_ revealed associations among loci in each population that did not disappear when removing clones and when considering MLLs (*p*‐value = 0.001) (Figure S6), especially in Ussisoo. However, these associations mostly affected different loci across populations, suggesting that they are not related to physical linkage among loci.

**TABLE 2:** Genetic diversity and differentiation in the two populations of *Cypripedium calceolus* analysed in this study. For SSRs, values for MLG‐based and the MLL‐based clone‐corrected data sets are identical when not reported otherwise.

Population	SSRs									SNPs					
	*H*_E_ (SE)		*H*_O_ (SE)		Private alleles (rarefied^a^)	Allelic richness^b^	*F*_IS_		*F*_ST_ (SE)	*H*_E_ (SE)	*H*_O_ (SE)	Private alleles	π (SE)	*F*_IS_ (SE)	*F*_ST_
	MLG‐based clone‐corrected data set	MLL‐based clone‐corrected data set	MLG‐based clone‐corrected data set	MLL‐based clone‐corrected data set			MLG‐based clone‐corrected data set	MLL‐based clone‐corrected data set							
Ussisoo	0.587 (0.071)	0.591 (0.070)	0.563 (0.079)	0.556 (0.078)	26 (1.86)	5.9	0.04	0.06	0.12 (0.03)	0.254 (0.0004)	0.241 (0.0005)	40,113	0.259 (0.0004)	0.059 (0.005)	0.10
Kõrgessaare	0.411 (0.059)	0.414 (0.059)	0.393 (0.058)	0.390 (0.058)	4 (0.51)	3.4	0.04	0.06		0.227 (0.0005)	0.214 (0.0006)	8695	0.232 (0.0005)	0.0497 (0.005)	

Abbreviations: π, average nucleotide diversity; *F*_IS_, inbreeding coefficient; *F*_ST_, fixation index; *H*_E_, expected heterozygosity; *H*_O_, observed heterozygosity; SE, standard error.

*Note*: Refer to Table S3 for the filtering strategies applied to the SNPs data set (“reduced”; genetic indices for SNPs are computed excluding sites significantly out of the Hardy–Weinberg proportions, after a false discovery rate correction for multiple comparisons).

^a^ Rarefied private alleles (private allelic richness in HP‐RARE) are based on 128 gene copies.

^b^ Allelic richness is based on the minimum sample size: 64 samples for the MLG‐based clone‐corrected data set and 57 samples for the MLL‐based clone‐corrected data set.

The analysis of genetic differentiation carried out in Structure showed that the most likely number of genetic clusters was *K* = 2 (Figure S7), with a few admixed individuals occurring in Kõrgessaare (Figure S8). We also report the results for *K* = 3, in which some internal structure emerges in Kõrgessaare (Figure S8).

### 3.2 Analysis of double‐digest RAD sequencing data

The results of the ddRADseq data analysis using the different filtering strategies are summarized in Table S3. In BayeScan, we detected two *F*_ST_‐outlier loci for which we did not recover any significant correspondence on the NCBI database (Figure S9), and we excluded them from the subsequent analyses (Table S3). For the estimation of genetic diversity parameters, we removed all possible clones (i.e., putative biological replicates) and samples with low coverage and a high percentage of missing data. This filtering strategy produced 34,484 polymorphic loci, with ~108,000 SNPs for Ussisoo and ~86,000 for Kõrgessaare (Table S3). After correcting the P‐values for the test of Hardy–Weinberg proportions, 77 SNPs significantly deviated from the Hardy–Weinberg proportions in Ussisoo and 184 SNPs in Kõrgessaare in the main data set (Table S3). Population‐level *F*_IS_ values were 0.06 in Ussisoo and 0.05 in Kõrgessaare (Table 2); at the locus‐level, most of the deviations from Hardy–Weinberg proportions were associated with positive *F*_IS_ values, suggesting a deficit of heterozygotes (results not shown). Removing the out‐of‐equilibrium sites, however, did not produce changes in the values of genetic diversity and differentiation. Genetic diversity indices for Ussisoo were slightly higher than for Kõrgessaare: *H*_O_ was 0.24 for Ussisoo and 0.21 for Kõrgessaare, *H*_E_ was 0.25 for Ussisoo and 0.23 for Kõrgessaare, and π was 0.26 for Ussisoo and 0.23 for Kõrgessaare (Table 2). The most marked differences between the two populations were observed in terms of private alleles (~40 K in Ussisoo and ~9 K in Kõrgessaare). The fixation index *F*_ST_ was ~0.1 (Table 2).

**FIGURE 3:**
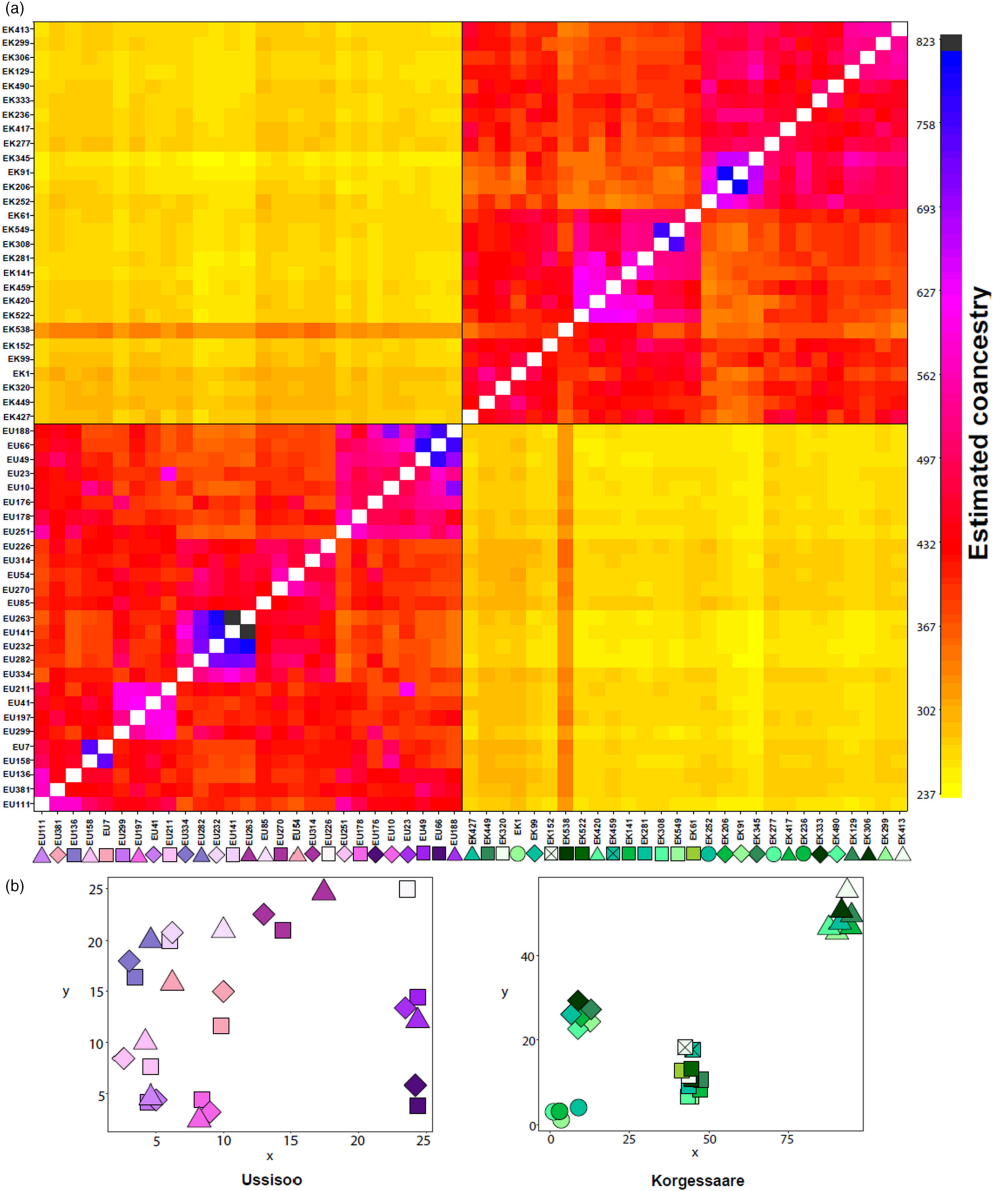
(a) Heatmap of the estimated coancestry between pairs of individuals obtained in fineRADstructure. Correspondence between colour gradient and coancestry value is indicated in the sidebar. Symbols located along the *x*‐axis in (a) indicate the spatial position of the genets as represented in the population maps (b).

Fine‐scale relationships as obtained in fineRADstructure revealed a clear differentiation between populations, with only one sample in Kõrgessaare (EK538) showing higher similarity with samples at Ussisoo (Figure 3). In Kõrgessaare, we observed a further population subdivision which reflected only partially the spatial distribution of samples. In Ussisoo, many sample pairs were highly related (with a coancestry coefficient sensu Malinsky et al., 2018 > 500). In general, most of the closest coancestry coefficients occurred between samples at short distances. The biological replicates EK549‐EK308 were among the sample pairs with the highest coancestry coefficients. However, six pairs (five in Ussisoo and one in Kõrgessaare) had a coancestry coefficient higher than the biological replicates, possibly because of missing data affecting samples differently (Figure S10; see also Gargiulo, Kull, et al., 2021). Among the sample pairs with the highest coancestry coefficients, none exhibited an identical MLG for the SSR loci (as opposite to EK549‐EK308, which exhibited an identical microsatellite genotype). In particular, each pair differed at four to seven loci, and most of the time they shared one allele at such divergent loci (the raw data is available on Dryad: https://doi.org/10.5061/dryad.6wwpzgn37).

### 3.3 Estimation of effective population sizes *N*_e_

Estimates of *N*_e_ obtained in NeEstimator differed somewhat according to the marker type used, especially in Kõrgessaare (Table 3). The analysis of the microsatellite data set (after excluding singletons) in Ussisoo produced an *N̂*_e_ equal to 16.9 (CI: 11–25.8) for the MLG‐based clone‐corrected data set and 20.6 (CI: 13.5–32.3) for the MLL‐based clone‐corrected data set. In Kõrgessaare, *N̂*_e_ was 24 (CI: 13.9–39.5) and 24.1 (CI: 13–42.9) when considering respectively the MLG‐based clone‐corrected data set and the MLL‐based clone‐corrected data set. When 16 admixed individuals were removed, *N̂*_e_ was 32.8 (CI: 18–59.5). In addition, accounting for the putative internal population structure (as observed for the second most‐likely *K* value, *K* = 3) determined different estimates: 6.1 (CI: 2.0–20.2) for the purple cluster (53 individuals) and 15.5 (CI: 8–28.7) for the blue cluster (74 individuals) (Figure S8; Table 3). Therefore, ratios of *N*_e_/*N*_C_ considering either the number of ramets or genets as an estimate for *N*_C_ were *N*_e_/*N*_ramets_ = 0.03 and *N*_e_/*N*_genets_ = 0.26 in Ussisoo, and *N*_e_/*N*_ramets_ = 0.04 and *N*_e_/*N*_genets_ = 0.13 in Kõrgessaare.

**TABLE 3:** *N̂*_**e**_ for the two populations of *Cypripedium calceolus* analysed in this study.

Population	SSRs										SNPs								
	MLG‐based clone‐corrected data set		MLL‐based clone‐corrected data set		MLG‐based no admixed individuals (K = 2)		MLG‐based data set for the purple cluster in *K* = 3 (no admixed individuals)		MLG‐based data set for the blue cluster in *K* = 3 (no admixed individuals)		*Data set “forNe” –* 27,136 SNPs			Median N̂_e_ on 40 subsets – ~800 SNPs			Without the admixed sample (EK538)		
	*N*	*N̂*_e_ (CI)	*N*	*N̂*_e_ (CI)	*N*	*N̂*_e_ (CI)	*N*	*N̂*_e_ (CI)	*N*	*N̂*_e_ (CI)	*N*	*N̂*_e_ (CI)	LD‐related bias corrected	*N*	median *N̂*_e_ (empirical CI)	LD‐related bias corrected	*N*	*N̂*_e_ (CI)	LD‐related bias corrected
Ussisoo	66	16.9 (11.0–25.8)	59	20.6 (13.5–32.3)	66	16.9 (11.0–25.8)	66	16.9 15.4 (11.0–25.8)	66	16.9 15.4 (11.0–25.8)	31	25.5 (15.8–49.1)	42.5	31	25.7 (21.1–29.3)	42.8	31	25.4 (15.8–49.1)	42.3
Kõrgessaare	191	24.0 (13.9–39.5)	157	24.1 (13.0–42.9)	175	32.8 (18.0–59.5)	53	6.1 (2.0–20.2)	74	15.5 (8.0–28.7)	31	5.7 (2.9–9.8)	9.5	31	5.9 (4.3–6.7)	9.8	30	5.2 (2.8–9.0)	8.7

*Note*: *N*, sample size; *N̂*_e_, effective population size estimate, calculated after excluding singletons (p‐crit depending on sample size). In parentheses, confidence intervals (CI) are obtained by jackknifing over loci, except when empirical CI are specified. Refer to Figure S8 for the Structure results (purple and blue clusters). Refer to Table S3 for the filtering strategies applied to the SNPs data set (only one SNP per locus was included in all NeEstimator analyses). Removing sites significantly deviating from the Hardy–Weinberg proportions did not produce any change in the *N*_e_ estimates. LD‐related bias correction refers to the formula 0.098 + 0.219 × ln(10) = 0.60, used to correct the potential bias caused by physical linkage among a high number of loci (see Waples et al., 2016): *N̂*_**e**_/0.60.

**FIGURE 4:**
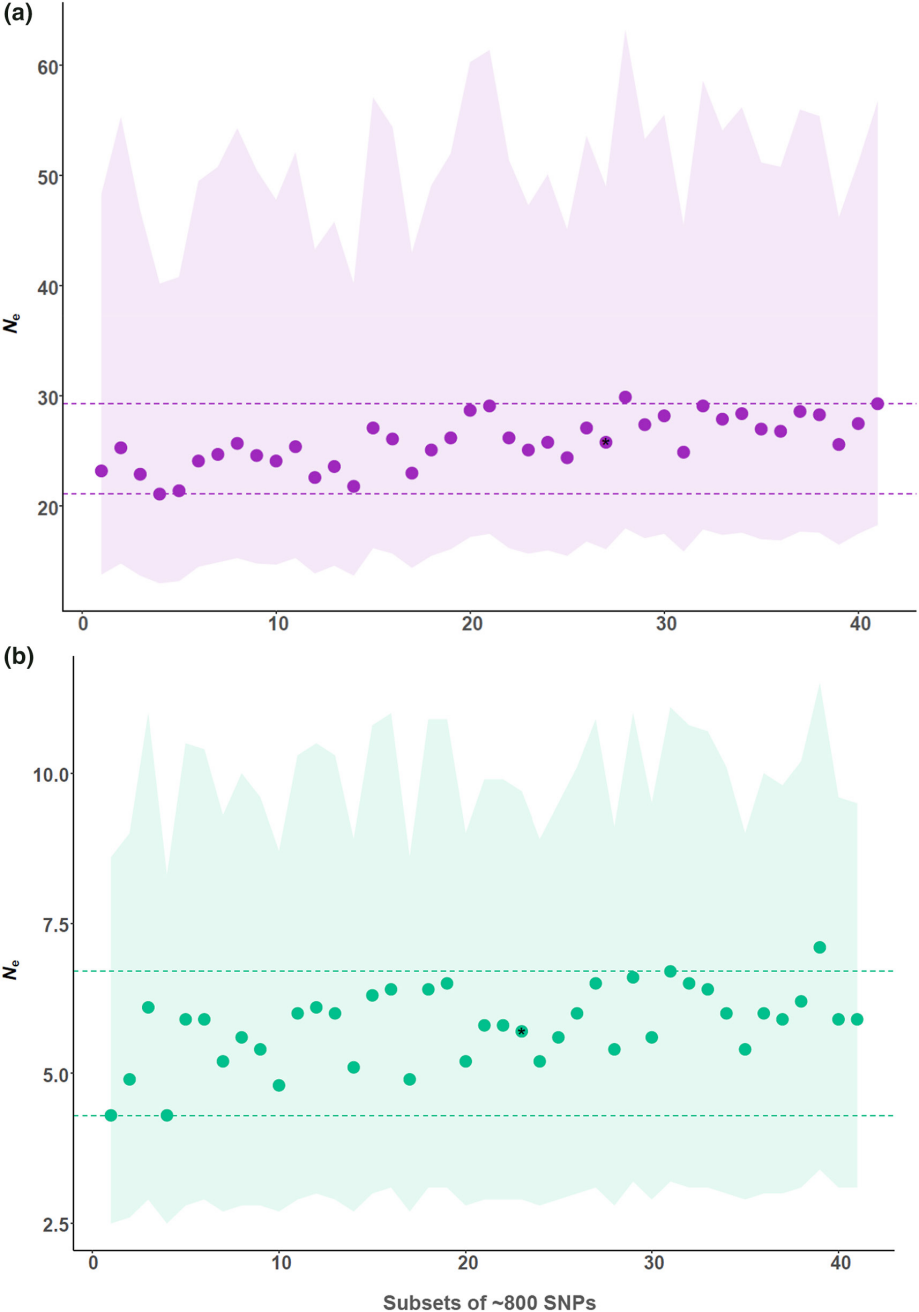
Estimates of the effective population size in the populations of *Cypripedium calceolus* at (a) Ussisoo and (b) Kõrgessaare, obtained from 40 different subsets of ~800 SNPs in NeEstimator (results reported in Table 3 and filtering strategies explained in Table S3). Dots indicate point estimates for *N*_e_, ordered by effective degrees of freedom, ranging between 560 and 941 in Ussisoo and 221 and 391 in Kõrgessaare. Shading indicates jackknife confidence intervals (Jones et al., 2016), whereas dashed lines indicate empirical 95% confidence intervals. Dots with asterisks indicate estimates obtained from the full data set (27,136 SNPs), included for comparison. Note the different scales of *y*‐axes for the two populations.

For the SNPs data set, Spearman's rank correlation rho showed a very weak positive correlation between the proportion of missing data and *F*_IS_ at each locus, for both Ussisoo and Kõrgessaare (rho = 0.108 for Ussisoo, rho = 0.087 for Kõrgessaare, *p* < 0.001), suggesting that although allele dropout may be the cause for positive *F*_IS_ values, nonrandom missing data do not strongly affect genetic indices and thus *N̂*_e_. The analysis of the total SNPs data set (after excluding singletons) produced an *N̂*_e_ equal to 25.5 (CI: 15.8–49.1) in Ussisoo and 5.7 (CI: 2.9–9.8) in Kõrgessaare. The latter estimate was still very small after removing the admixed sample EK538: 5.2 (CI: 2.8–9.0) (Table 3). We also evaluated how precision changed, depending on the LD among different loci, and reported the results obtained by analysing 40 different subsets of 800 loci in NeEstimator (Figure 4). In Ussisoo, the analysis of the 40 subsets (after excluding singletons) produced a median *N̂*_e_ equal to 25.7 (empirical CI: 21.1–29.3), whereas in Kõrgessaare, the analysis of the 40 subsets produced a median *N̂*_e_ equal to 5.9 (empirical CI: 4.3–6.7) (Table 3). For all estimates based on SNPs, a 40% downward bias due to physical linkage among loci can be expected in a species with 10 chromosomes (haploid number), according to the formula in Waples et al. (2016), although we expect that using only the first SNP in the data set was already mitigating some of the downward bias (Table 3).

## 4 DISCUSSION

### 4.1 Effective population size (*N*_e_) in partially clonal plants

Our study represents the first exhaustive comparison among the number of ramets, number of genets, and genetic contemporary estimate of *N*_e_, based on two molecular marker types (microsatellites and ddRADseq‐SNPs). Our findings show that *N*_e_ in a partially clonal plant cannot reliably be predicted based on the number of genets because demographic events (e.g., bottlenecks or declines) strongly influence *N*_e_.

In particular, we observed that despite the smaller number of ramets and genets in Ussisoo, and its lower rate of sexual reproduction, this population has higher genetic diversity and higher *N̂*_e_ than Kõrgessaare, and this is consistent with the stable demography of Ussisoo over the years. In Kõrgessaare, contemporary *N̂*_e_ is extremely small when computed on the SNPs data set; considering the long generation time of this species: *T* = *α* + [*s*/(1 − *s*)] = 30 years [according to the formula in Lande et al. (2003), where *T* is the generation time in years, *α* is the age at first flowering (6 years), and *s* is the adult survival rate, which was estimated as ~0.96 (Shefferson et al., 2012)] and the fact that only adult individuals were included in the SNPs data set, *N̂*_e_ still maintains the signature of a strong population bottleneck (which occurred during the founder event, probably less than 100 years ago). Nevertheless, this population has recently undergone an expansion, and this is also suggested by the larger *N̂*_e_ obtained from the microsatellites data set including juveniles.

The differences between Ussisoo and Kõrgessaare show that generalization at the species level is not possible without a full understanding of the demographic changes different populations undergo, and that reproductive patterns alone cannot explain a small *N*_e_. In different species, differences among *N*_e_/*N*_C_ ratios can be dictated by generation length and age at maturity (the longer these are, the more *N*_e_ increases) and lifetime variance in reproductive success (higher variance decreases *N*_e_) (Frankham, 1995; Nunney, 1991, 1993; Waples, 2016a). Within the same species, with generation length and age at maturity being similar, differences in *N*_e_ among populations will especially depend on variance in reproductive success and demographic events.

In terms of variance in reproductive success, larger clumps (genetic individuals with more ramets, which are not always older than other genetic individuals) may sexually reproduce more than smaller clumps, and may also inbreed via geitonogamy (i.e., mating among ramets of the same clone). These factors may reduce *N*_e_ by favouring larger individuals with more flowers and by causing inbreeding, respectively. The behaviour of pollinators may partially prevent geitonogamy, as pollinators tend to abandon clumps when they discover sexual deception or food deception in orchids that do not offer such rewards (Jersáková et al., 2006; Tremblay et al., 2005; Whitehead et al., 2015), but this may not be the case in other species. Moreover, the advantage of larger or older clumps may be reduced if some of the ramets die over the years, and this would counterbalance the reduction of *N*_e_ via lifetime variance in reproductive success. Another phenomenon that could potentially counterbalance the reduction of *N*_e_ via lifetime variance in reproductive success is vegetative dormancy (Davison et al., 2013; Lesica & Steele, 1994; Shefferson et al., 2001, 2020), which may cause some plants to skip breeding in some years, spreading lifetime reproductive success among individuals (Waples & Antao, 2014).

### 4.2 Factors affecting estimates of effective population size (*N̂*_e_)

Biological and technical factors may strongly influence the estimation of *N̂*_e_ (Frankham, 2019; Luikart et al., 2010; Palstra & Ruzzante, 2008). For example, gene flow and population structure can have strong confounding effects on *N̂*_e_ (Luikart et al., 2010; Waples & England, 2011). Although the long lifespan of *C. calceolus* implies more opportunities for migration to have an impact on the patterns of LD, ecological observations in orchids suggest that local recruitment, especially at short distances from parental plants, predominates over recruitment of nonlocal seeds (Chung et al., 2009; Duffy et al., 2020; Hedrén et al., 2021; Jacquemyn et al., 2009; Zhang et al., 2019), and that long‐distance dispersal, although important in the colonization stage, contributes less to gene flow once a stable population has been established (Hedrén et al., 2018). Such observations are corroborated by our genetic results, as we found clusters of similar genets in Ussisoo (Figure 3) and most juveniles were related to local adults in Kõrgessaare (Figure 2b). Therefore, we believe migration does not substantially influence our *N̂*_e_ (see also Table 3), although some internal population structure may influence *N̂*_e_ in Kõrgessaare (Figure S8).

The influence of pseudoreplication on confidence intervals is also important when estimating *N*_e_ for species of conservation concern, because it may lead to adopting measures that are too severe if confidence intervals are much narrower than the true ones (Waples, 2021; Waples et al., 2021). We investigated the influence of pseudoreplication (i.e., the lack of independence among thousands of loci, as they occur within a much smaller number of chromosomes) on *N̂*_e_ confidence intervals by evaluating the differences in precision between a Snip data set including ~30 K SNPs and subsets of 800 SNPs (Waples et al., 2021). The empirical CI across the subsets of 800 loci was narrower than the jackknife CI, and this is because empirical CIs do not account for the uncertainty associated with sampling individuals, whereas jackknife CIs do (Waples et al., 2021). Regardless, results were consistent and point *N̂*_e_ were almost identical between the two sets, showing that *N̂*_e_ and their confidence intervals did not depend on the subsample of loci included in the analysis. A similar analysis performed by Moran et al. (2019) produced much narrower empirical CIs than jackknife CIs, which extended to positive infinite values.

Statistical biases may also arise as a result of mixed‐age adult sampling, which may cause a 10%–50% underestimation from microsatellites and from SNPs (Waples et al., 2014). Age‐structure may also be the cause for the Wahlund effect and the discrepancy between microsatellite and SNP estimates observed, especially in Kõrgessaare, where young individuals were abundant and were included in the SSRs data set but not in the SNPs data set. Such observations are particularly important as they suggest that adults in Kõrgessaare show a strong signal of the founder effect, but this signal largely disappears when offspring of recent reproduction are sampled. This also shows, once again, the importance of sampling strategies for both study design and interpretations.

### 4.3 *N*_e_/ *N*_C_ ratios and implications for conservation

Although a small *N*_e_/*N*_C_ ratio may be perceived as a typical feature of orchids, even in the absence of clonal reproduction (Trapnell et al., 2022; Tremblay & Ackerman, 2001; Tremblay et al., 2005), and the equilibrium among life‐history traits can buffer genetic drift, populations are still susceptible to environmental and genetic stochasticity (Palstra & Ruzzante, 2008). In the terrestrial orchid *Cremastra appendiculata*, Chung et al. (2004) concluded that genetic diversity is maintained despite a small local *N*_e_, possibly because of metapopulation dynamics or because genetic diversity reflected past levels of diversity, whereas their *N̂*_e_ reflected a contemporary estimate.

Similarly, we observed that indices of genetic diversity (e.g., heterozygosity) contrast with the *N̂*_e_ found (see also Siol et al., 2007). In particular, *N̂*_e_ is significantly smaller than the thresholds signalling critical genetic erosion (*N*_e_ < 50; Jamieson & Allendorf, 2012; Frankham et al., 2014). In Kõrgessaare, we have explained the extremely small *N̂*_e_ as the legacy of a founder effect and, if the recent population expansion continues, *N*_e_ will probably increase in a few generations and the genetic signal of the founder effect will disappear. In Ussisoo, it is possible that moderate levels of genetic diversity despite small *N̂*_e_ persist because of the long lifespan of genets, and thus reflect past levels of gene flow. Such time lags between the occurrence of demographic changes and the attainment of new equilibrium values of genetic parameters (Epps & Keyghobadi, 2015) are also known in the literature as “genetic extinction debt” (Honnay et al., 2006; Vranckx et al., 2012) and mostly depend on life‐history traits. In the partially clonal species *Arnica montana*, Van Rossum and Raspé (2018) observed that populations maintain high genetic diversity (at microsatellite loci) despite a small population size, because of genet longevity, inbreeding depression in early development stages, or no recruitment. Although they did not estimate *N*_e_, they recognized that in such conditions, the number of ramets misrepresents the conservation status of populations. We argue that estimating contemporary *N*_e_ is crucial for evaluating the conservation status of a population when there is a time lag between the occurrence of a perturbation and the appearance of a measurable response in the genetic structure of the population.

Very few reliable *N*_e_/*N*_C_ estimates are available for plants based on genetic data over multiple generations (Frankham, 2019), and none of these plants have life history traits relatable to *C. calceolus* (e.g., Siol et al., 2007). We found the ratio *N*_e_/*N*_ramets_ <0.05 in the two populations of *C. calceolus*, whereas the ratio *N*_e_/*N*_genets_ was <0.3 (Table 3, when considering the estimates based on microsatellites, as SNPs were analysed only in a subset of samples). We assume that estimating *N*_e_/*N*_C_ ratios using the number of genets (ideally flowering genets to avoid including juveniles and immature individuals) as a surrogate for the number of mature individuals in a population is more appropriate than the number of ramets for comparisons among species. Obtaining multi‐generational *N*_e_ estimates in a species with a long generation time, such as *C. calceolus*, remains challenging, but our results may be used for this purpose if complemented with data available in the future. Including populations from further sites that may have different demographic histories and balance between clonal and sexual reproduction may also offer a more robust picture of *N*_e_ in this species.

In summary, we used a single‐sample, contemporary estimate of *N*_e_ to understand how effective population size changes depending on the balance between clonal and sexual reproduction. We provided the first exhaustive comparison between the number of ramets, number of genets, and contemporary *N̂*_e_. The *N*_e_/*N*_ramets_ and *N*_e_/*N*_genets_ ratios we provide may be used as reference points by researchers and practitioners interested in the magnitude of these ratios in their partially clonal species of interest, and in general for meta‐analyses across species (Frankham, 2019, 2021).

As *N*_e_ is notoriously difficult to calculate, we considered the factors potentially affecting our estimates, and we showed how estimates can differ when using different molecular marker types and different sampling strategies. In addition, the influence of pseudoreplication on confidence intervals concerns modern data sets regardless of the study species, and we showed that *N*_e_ point estimates obtained from ~30 K loci and from subsets of ~800 loci were comparable despite slightly narrower confidence intervals for the larger data set.

Most importantly, we found that effective population size in partially clonal plants cannot be predicted based on the number of genetic individuals (or genets) because demographic events (i.e., changes in the number of individuals over time) strongly influence *N*_e_ and this influence can last for decades, depending on the generation time of the species and other life‐history traits. Our findings are especially relevant in partially clonal species of conservation concern, in which population declines may not be detected by only counting individuals or by only ascertaining the number of genets using genetic methods. Estimating contemporary effective population size in partially clonal species is therefore crucial for evaluating their conservation status, and results should be interpreted in light of population‐specific demographic changes over time.

## CONFLICT OF INTEREST

The authors declare no competing interests.

## BENEFIT‐SHARING STATEMENT

Benefits from this research accrue from the sharing of our data and results on public databases as described above.

## Supporting information


Supporting information S1.

## Data Availability

Double‐digest RAD sequencing raw data are available under the BioProject PRJNA682386 on National Center for Biotechnology Information (NCBI). Microsatellite multilocus genotypes and filtered SNPs data are available on Dryad at: https://doi.org/10.5061/dryad.6wwpzgn37.
