# Supplementary material for: Effective population size in a partially clonal plant is not predicted by the number of genetic individuals
Source: Evol Appl. 2023 Feb 21;16(3):750–66. doi: 10.1111/eva.13535 (PMC10033856; doi:10.1111/eva.13535)
Supplement: Supplementary file 1 — Supporting information S1. [file EVA-16-750-s001.docx]

Supplementary Material to **“Effective population size in a partially clonal plant is not predicted by the number of genetic individuals”** by Roberta Gargiulo, Robin S. Waples, Adri K. Grow, Richard P. Shefferson, Juan Viruel, Michael F. Fay, Tiiu Kull. Published in Evolutionary Applications.


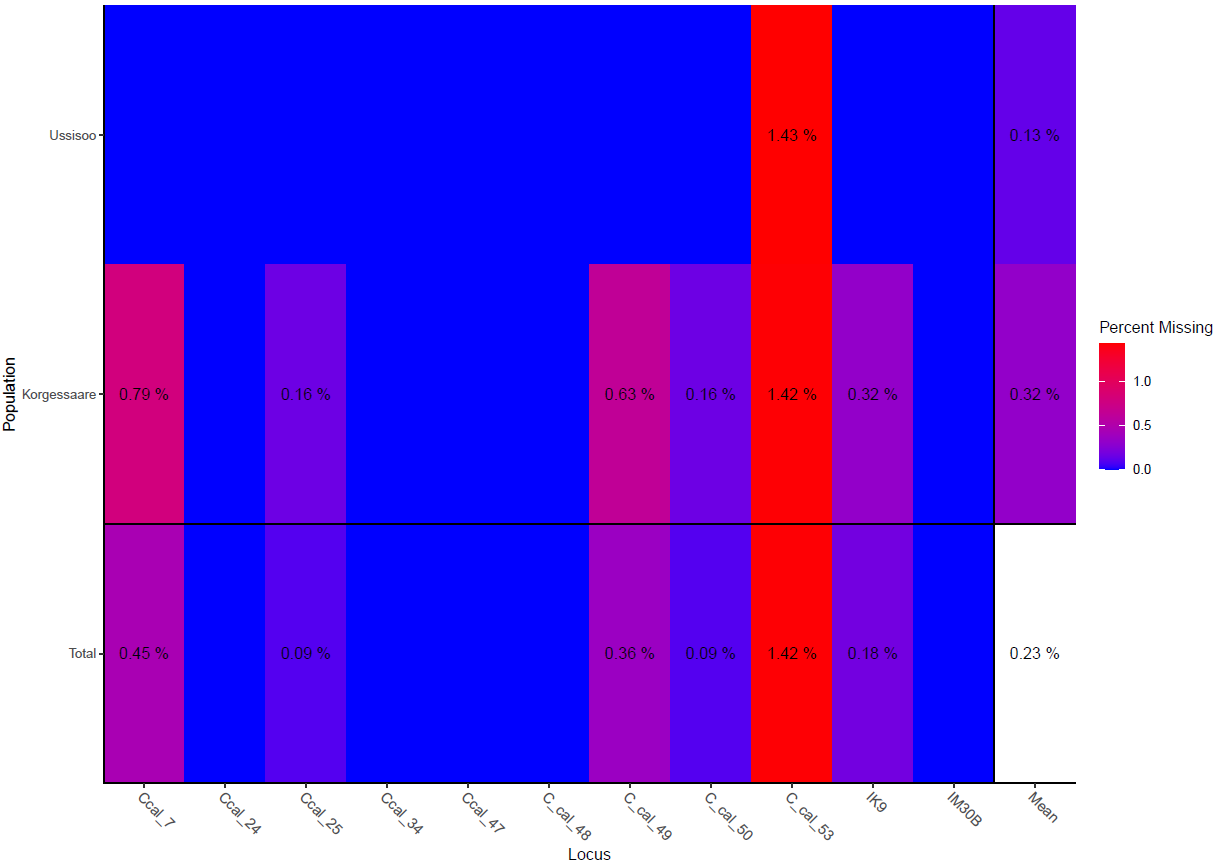


Figure S1. Missing data in the SSR data set of *C. calceolus* for Ussisoo and Kõrgessaare*.*


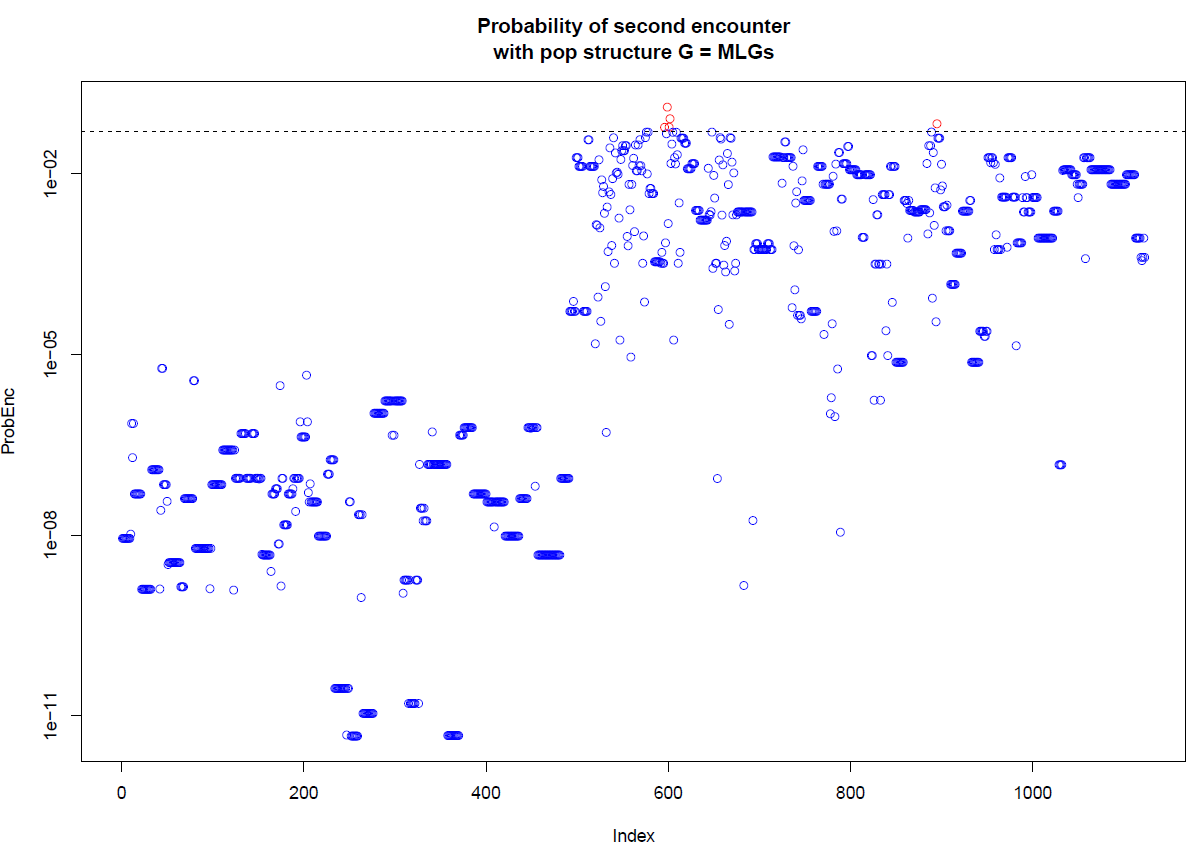

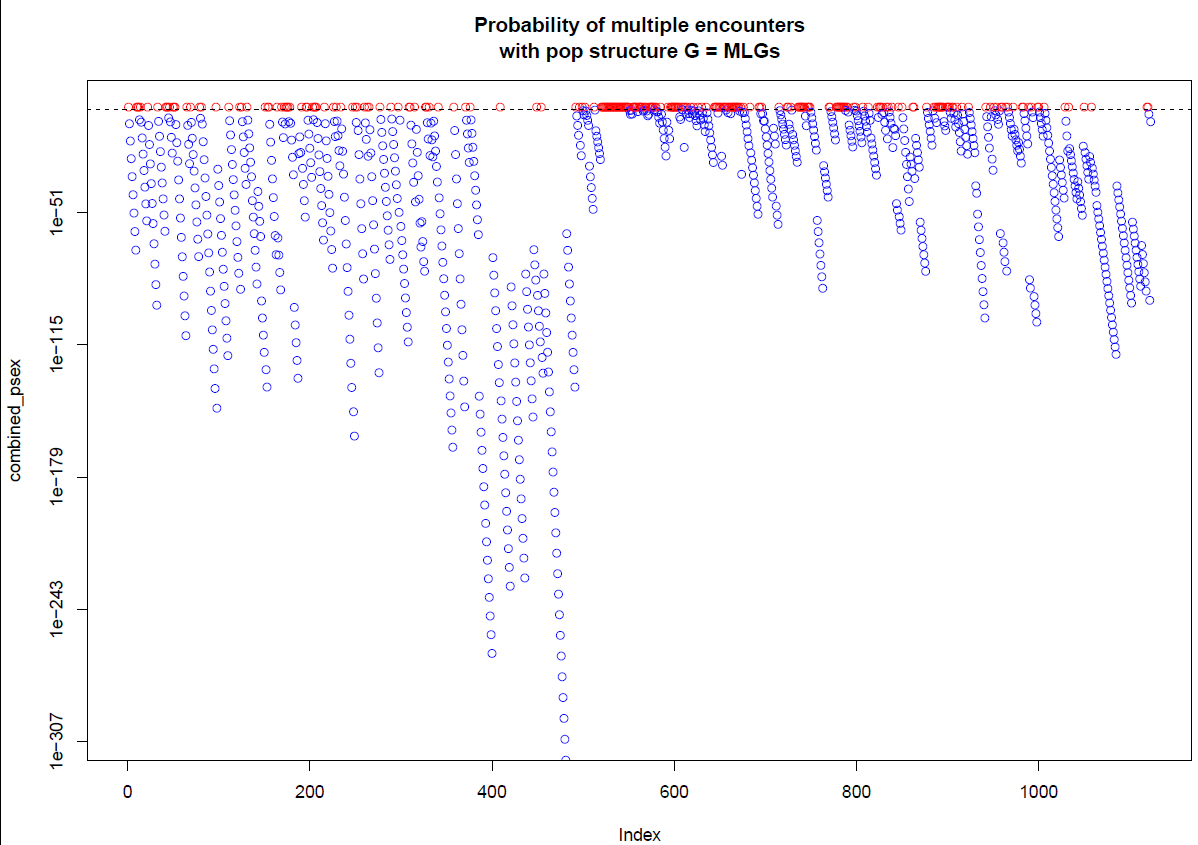


(b)

(a)

Kõrgessaare

Ussisoo

Kõrgessaare

Ussisoo

Figure S2. (a) Probability of encountering a second multilocus genotype identical by chance (*psex* with “single” method; Parks & Werth, 1993); (b) *psex* for multiple encounters (*psex* sensu Arnaud-Haond et al., 2007).


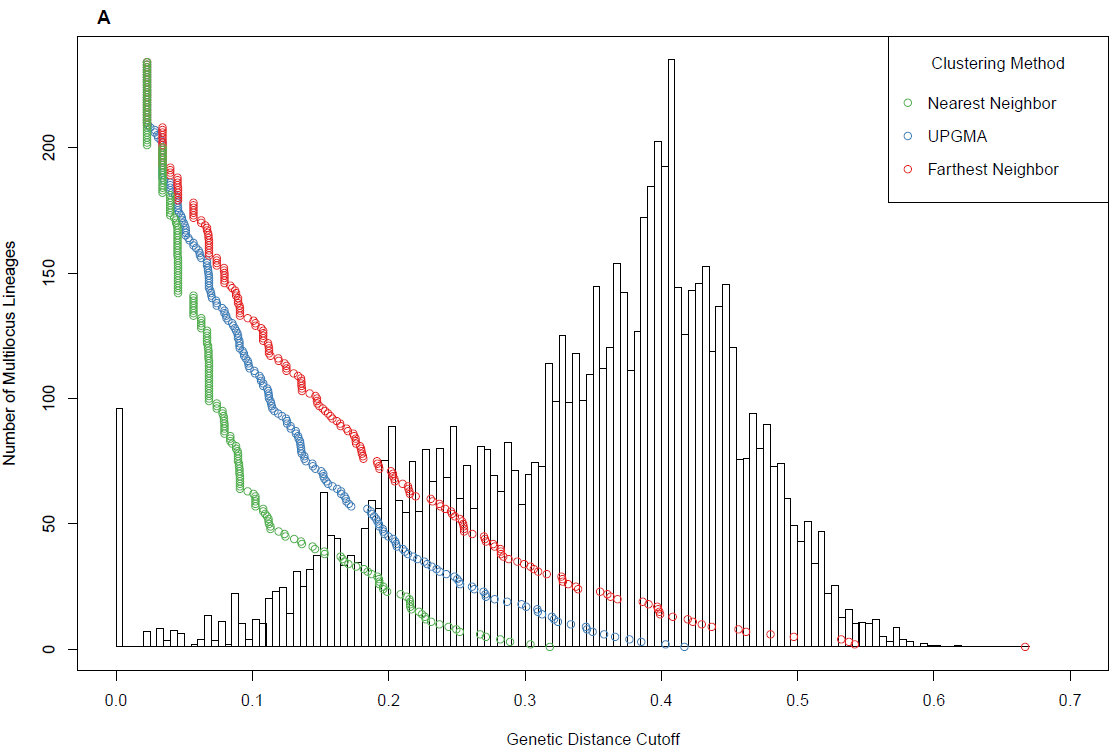

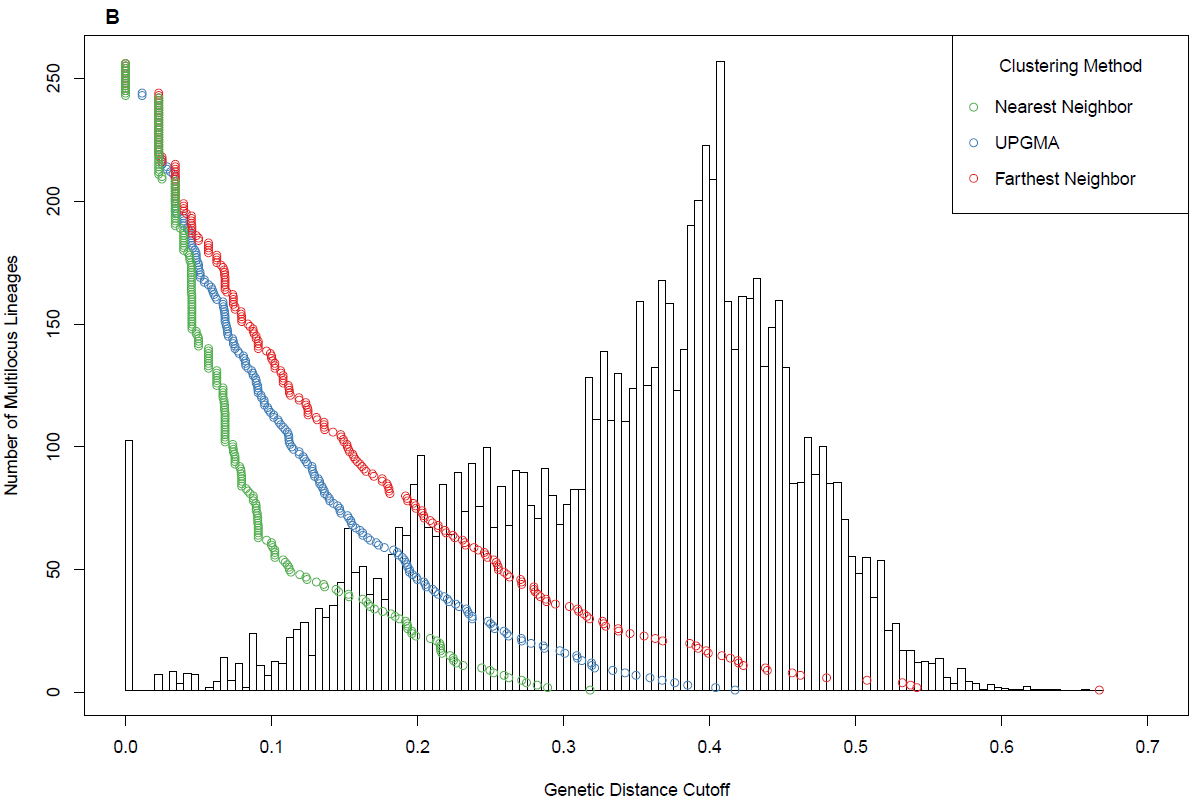

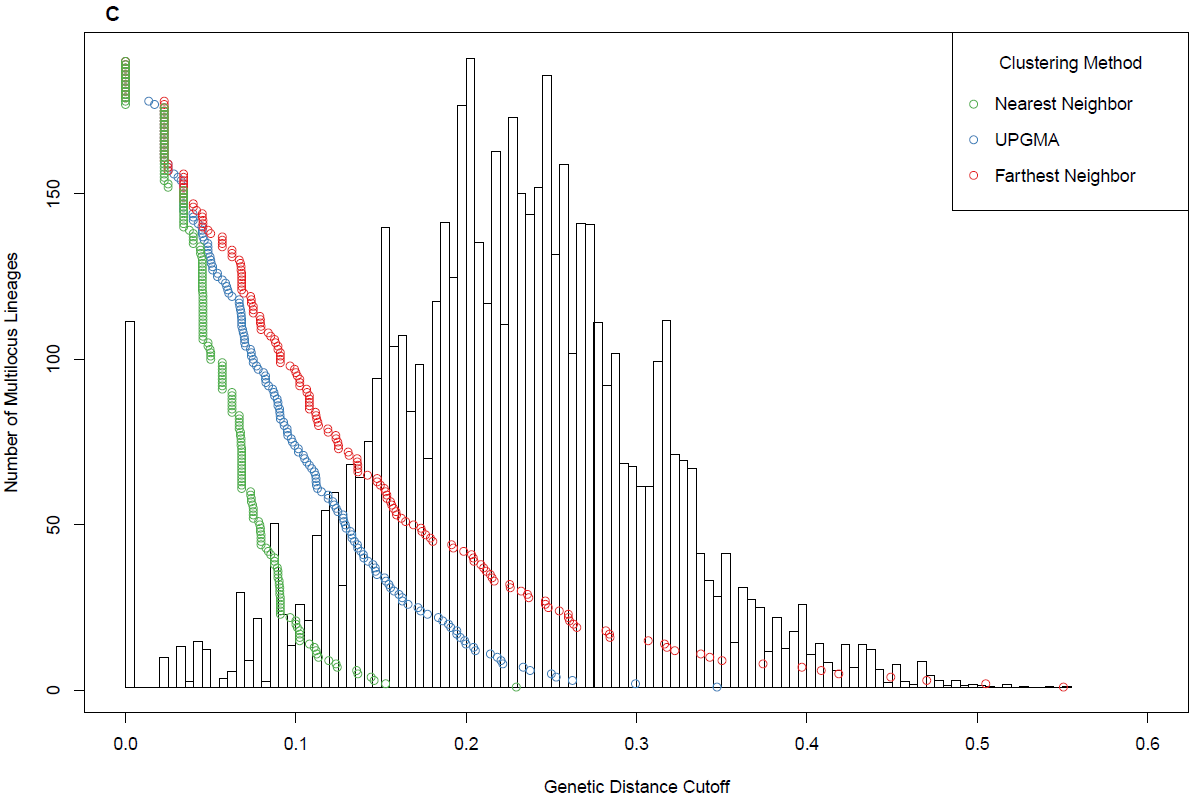

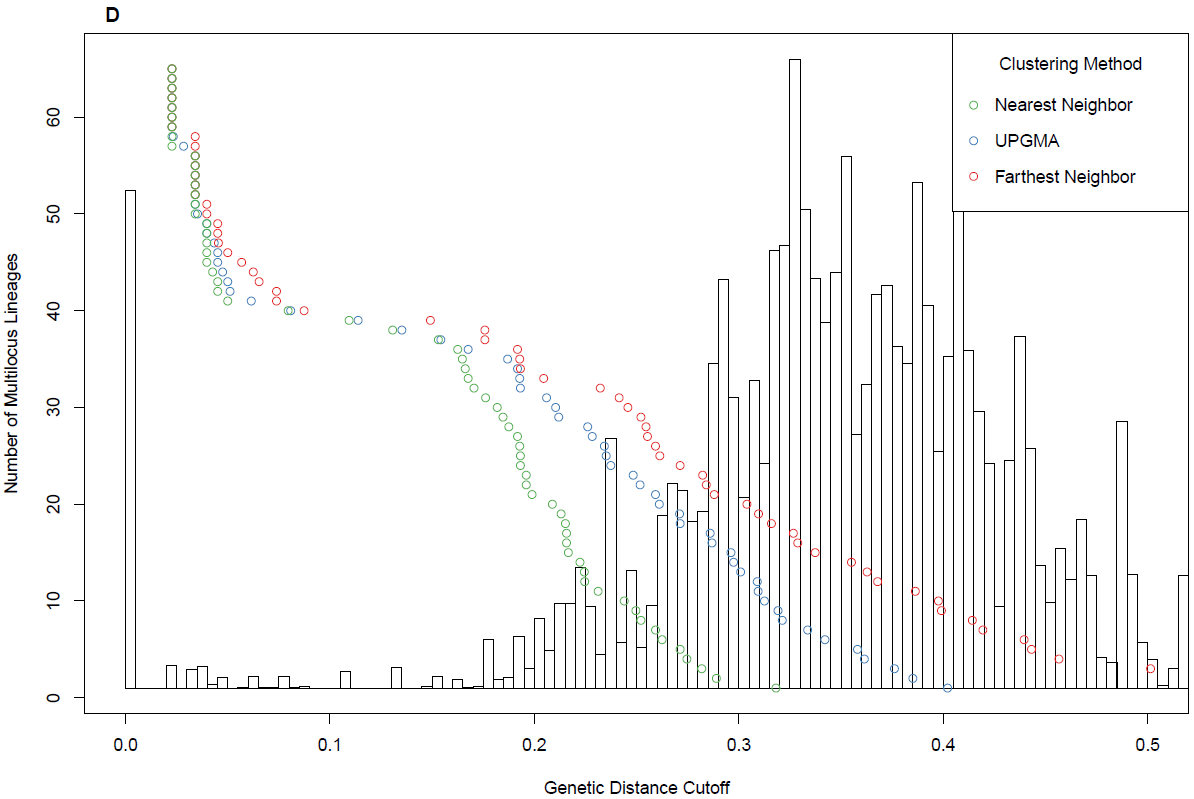


(d)

(c)

(b)

(a)

Figure S3. Genetic-distance thresholds for collapsing multilocus genotypes (MLGs) into multilocus lineages (MLLs). We treated missing data in two ways: (a) keeping them in the data set by using the option *missingno = “asis”*, and (b-c-d) discarding the genotypes with a higher percentage of missing data (28 genotypes contained missing values > 5%). The analyses were also run separately for (c) Kõrgessaare and (d) Ussisoo.


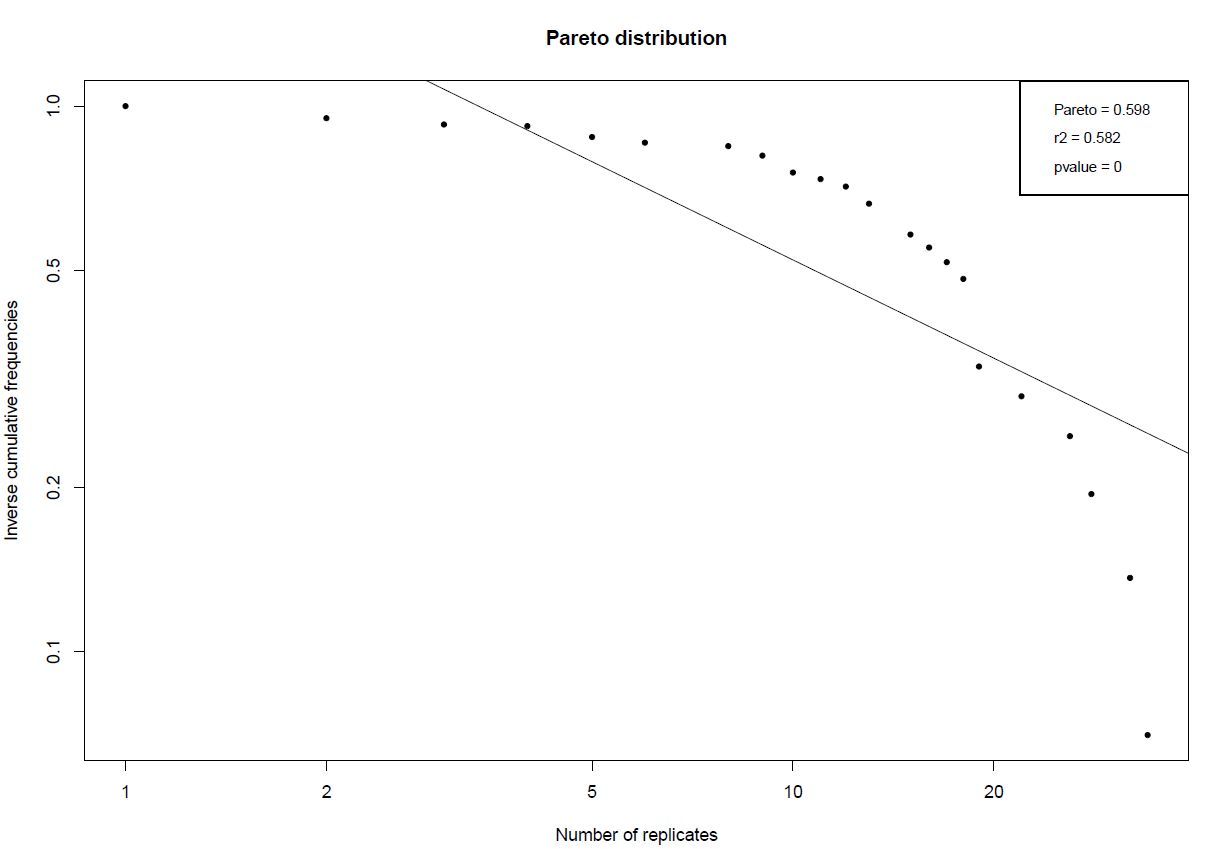


(a)

(b)


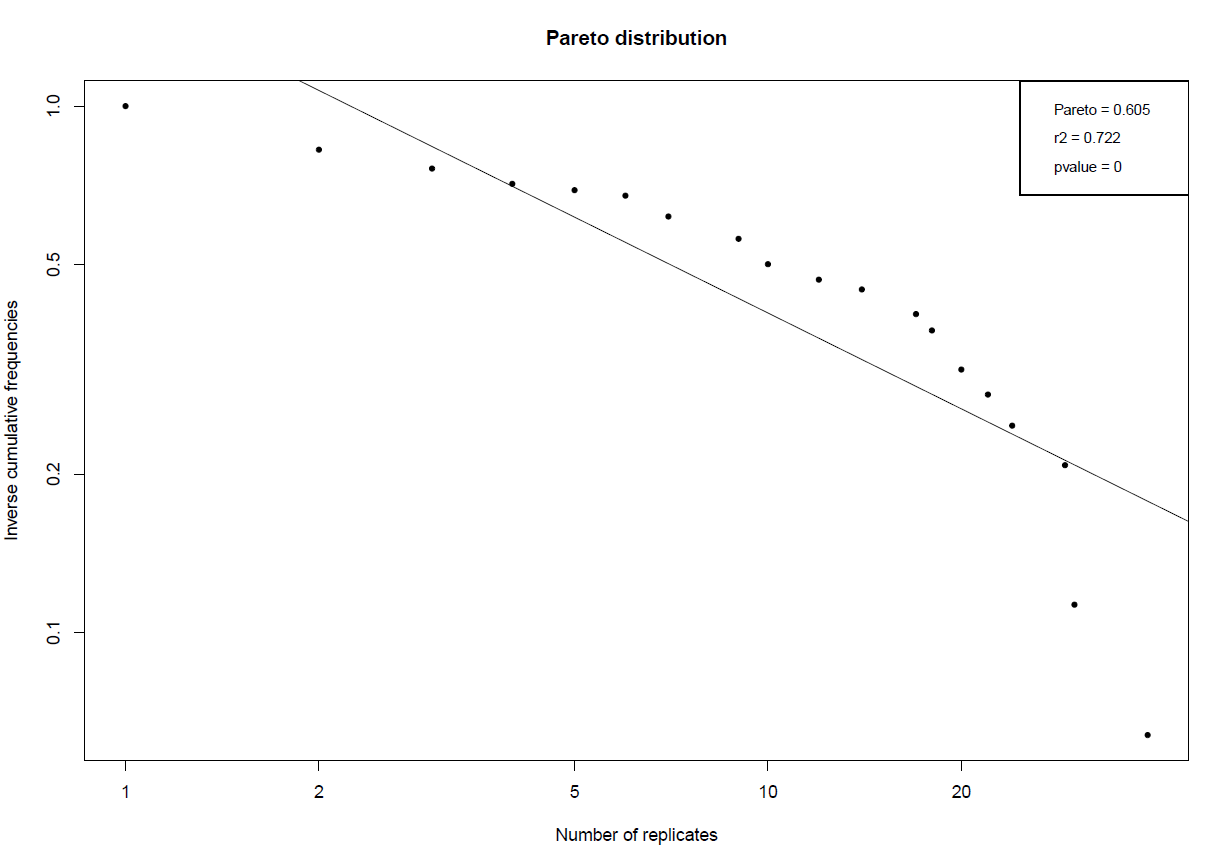


Figure S4. Pareto distribution for (a) Ussisoo and (b) Kõrgessaare.


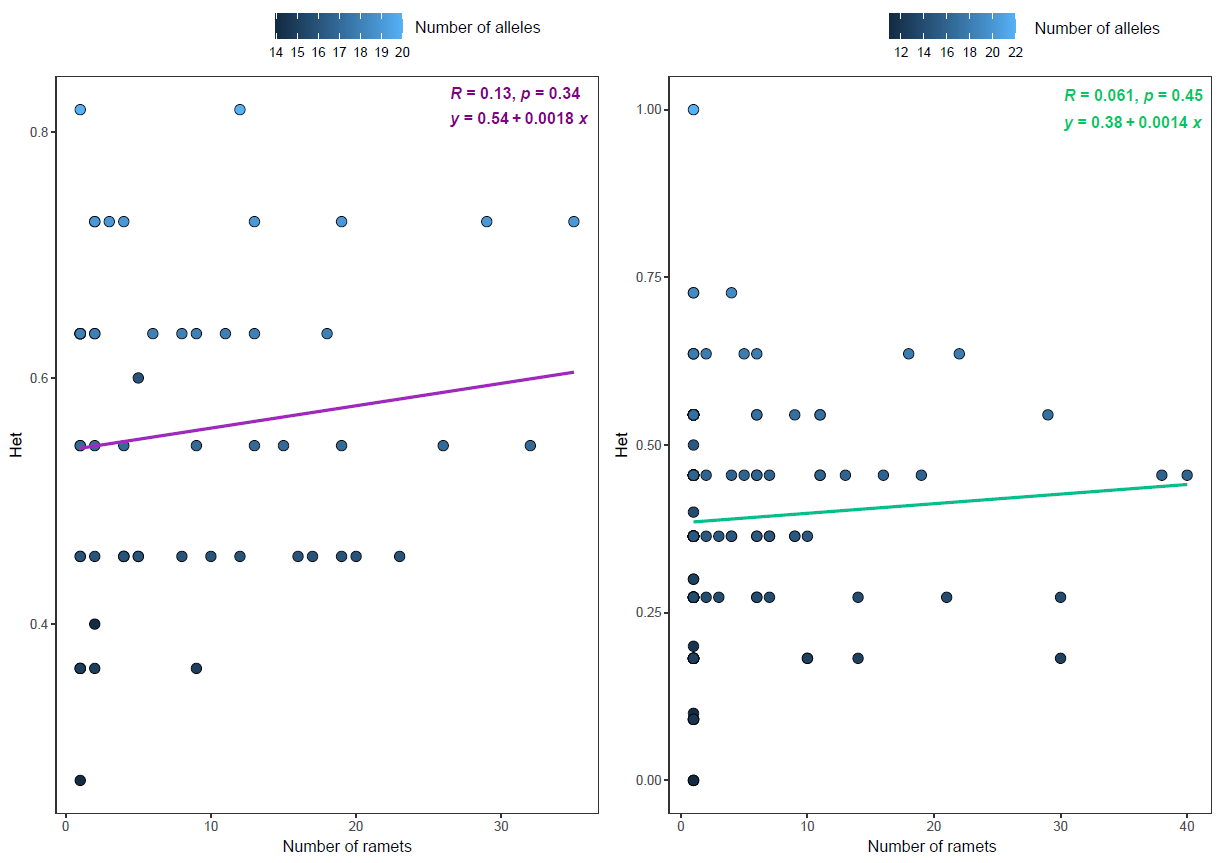


(b)

(a)

Figure S5. Correlation between clump size and heterozygosity in (a) Ussisoo and (b) Kõrgessaare. Notice that the correlation in Kõrgessaare may be biased because of the high number of juveniles included in the data set, which may not reach maturity.

(b)

(a)


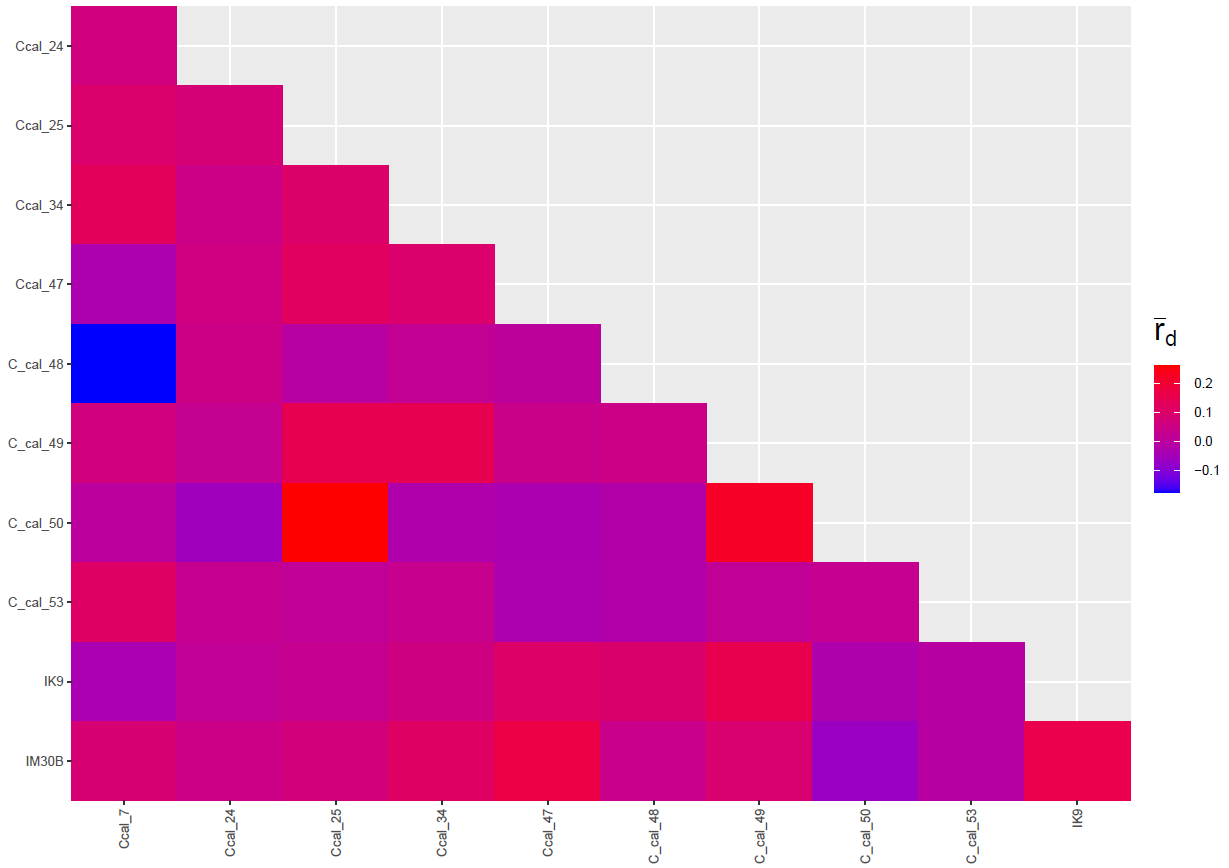

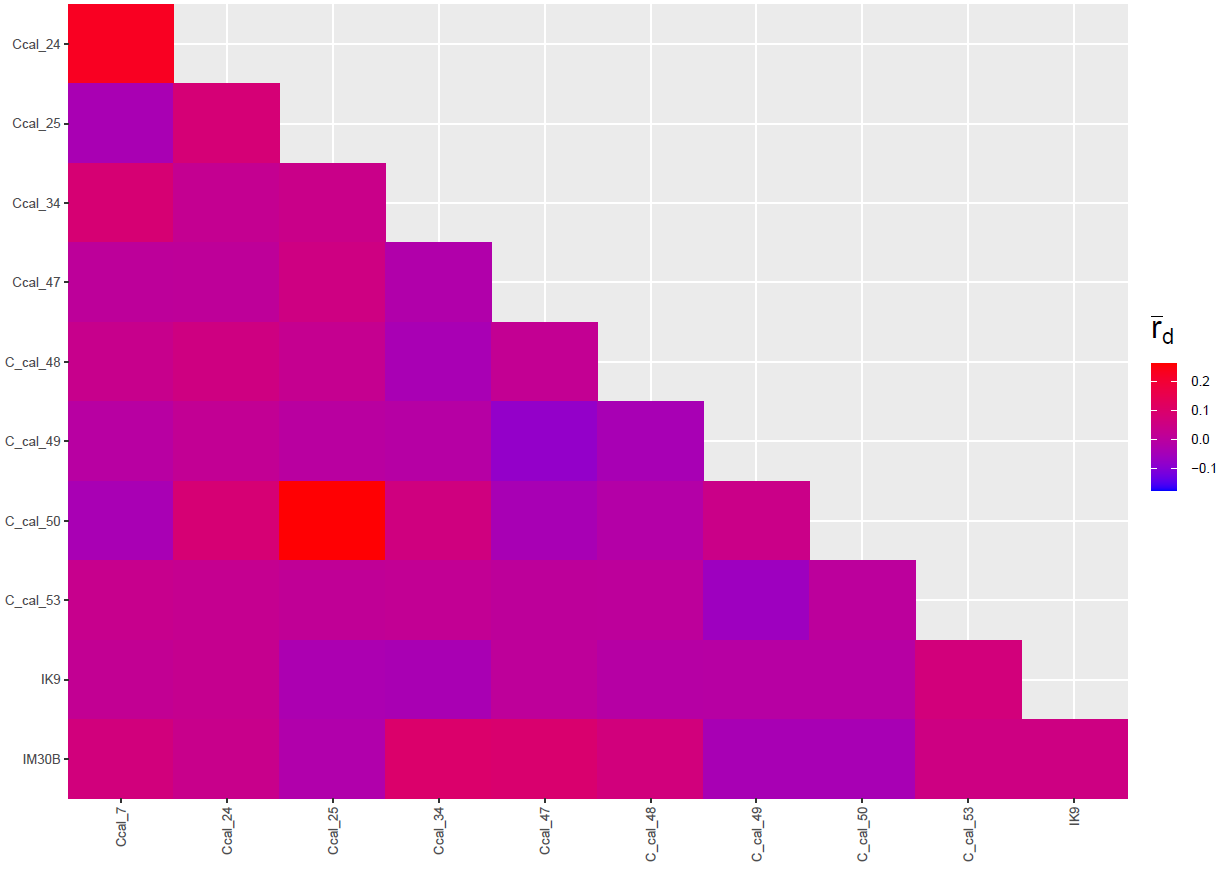


Figure S6. Heatmaps of the index of association (r_d_) among loci in (a) Ussisoo and (b) Kõrgessaare, with warmer colours indicating a higher r_d_.


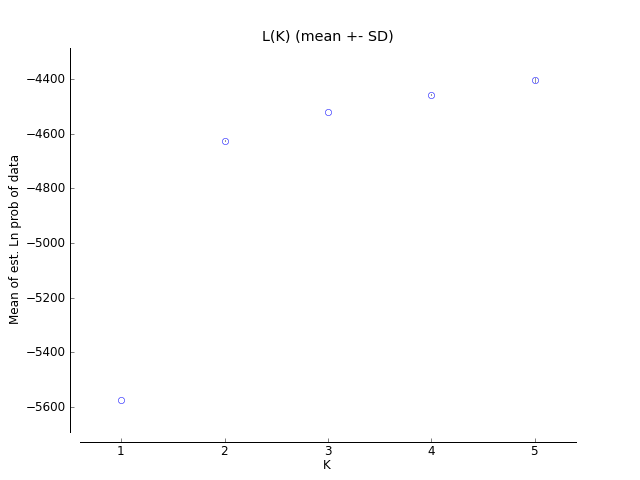

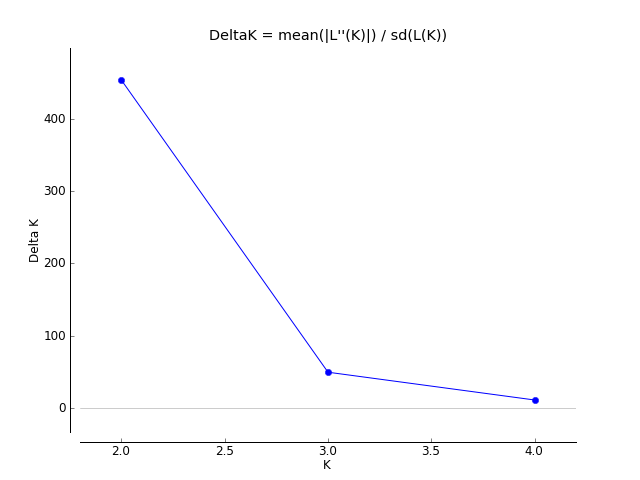

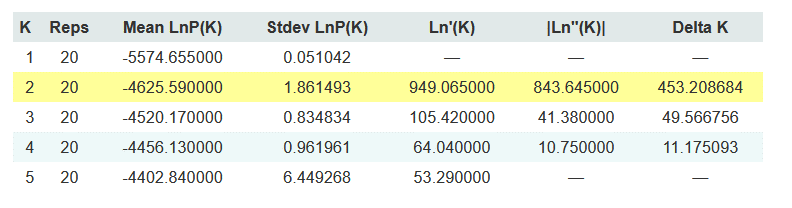


(a)

(b)

Figure S7. Results of the (a) LnPr(*X*|*K*) method and of the (b) Δ*K* Evanno method for the evaluation of the most likely *K,* and Evanno table, as obtained for the microsatellite data set of *C. calceolus* in Structure Harvester.


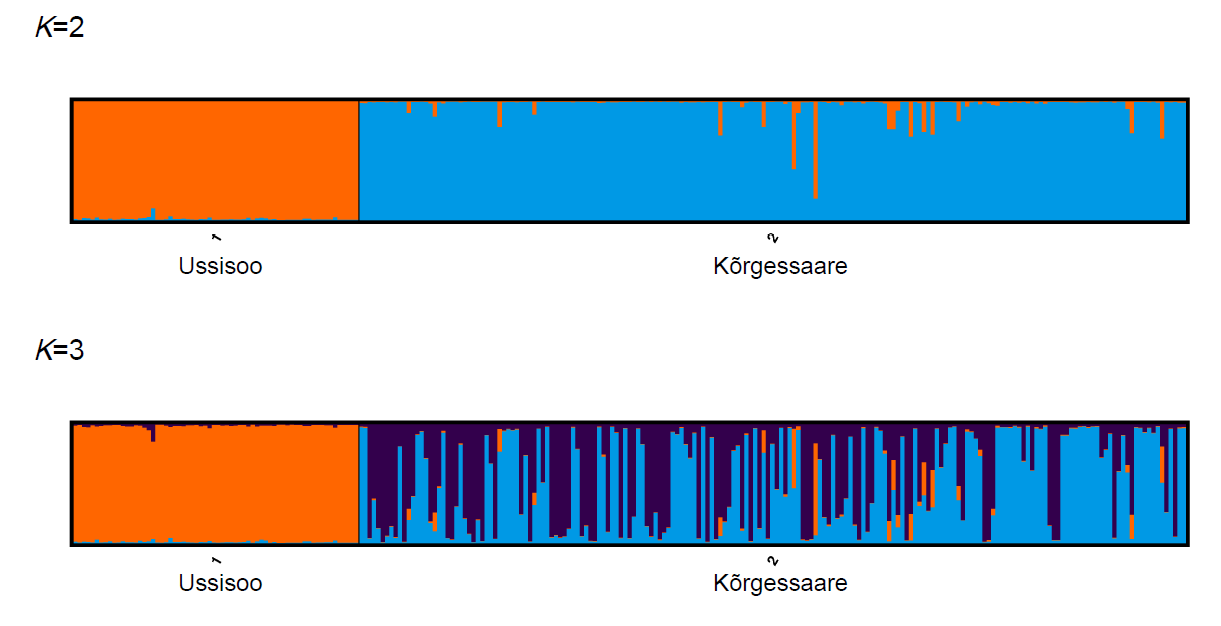


Figure S8. Bar plots for the results of the Structure analysis on the microsatellite data set of *C. calceolus*, as obtained in CLUMPAK.

K = 2 is the most likely K value, and K =3 is shown for comparison.


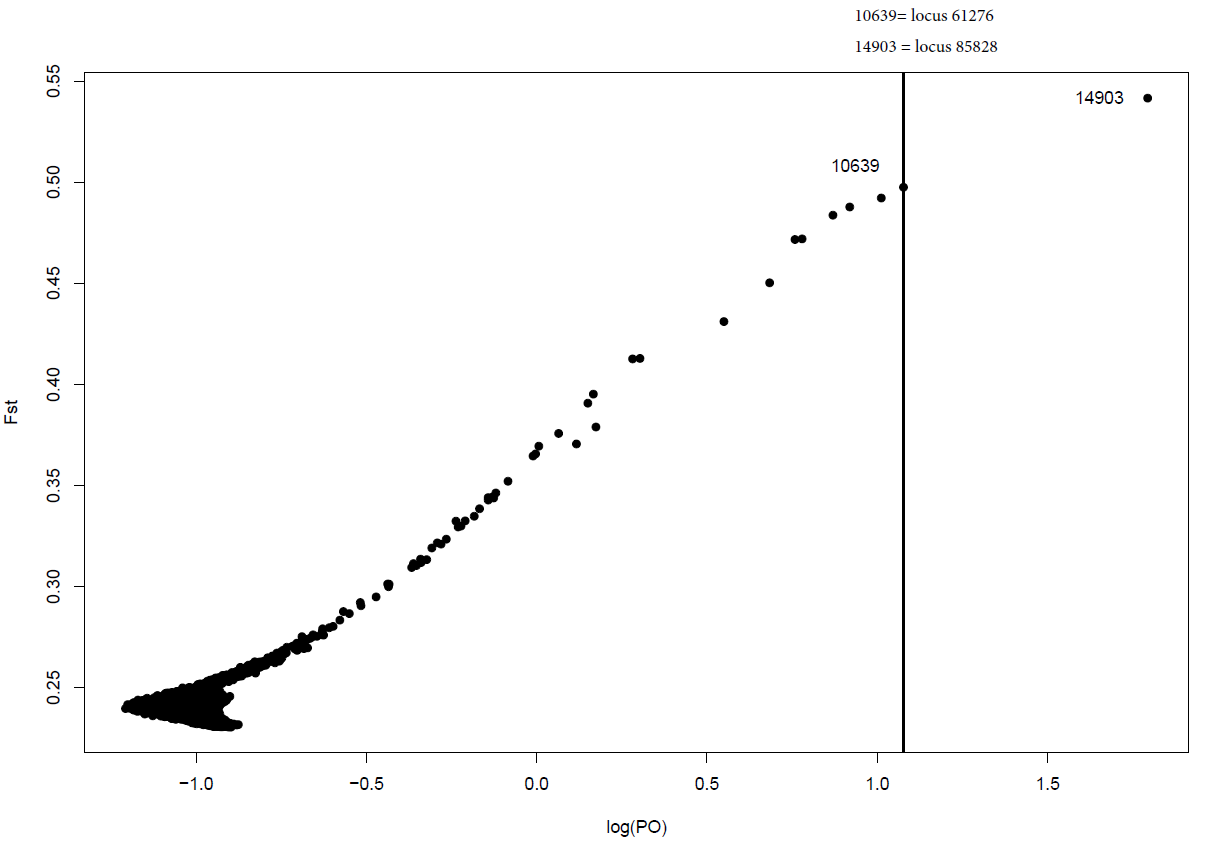


Figure S9. Results of the BayeScan analysis showing two potential outlier loci:

Locus 61276

AATTCACTTTTCATTCTTGGGAGAGCATTTGGGTCATTGACCTAAGCACATTGTAGAGACATCTTCCCCAACCTACCTAGTGGGCTATGGGTTGCAATCATCTCATATCCATACTTGGTGCATCTTATGCACGATTTTGCATTGTTATGAATACACCATCCCGACATTTATTCCTCAAACACCCCTAATGCTATAGATGTTGATTCTTTGAGGATGAAGGAGGACATGGTCCTCAATCATAGGGTTCCTCCCGGGTTAAACCCGTGAATCTCCAAACCATGTGGCCCGTAAGTGAAGGAGTCTTCCATGCCAGGCATAGAGCACACACCCAAGGTTATGTTTGTTGGGGCTAATGAGAGTCAACCCG

Locus 85828

AATTCTCAGTGAAGAATGGTATTGGAGAGAGAAGAAGTTTTCTTCACCATCATTGGAGAAGAATCCCAAGAAAAATCCCTTTATTTTCTATCTTTCACTACTTAAAATAGGAGGAAATTTATGTTCCCCATGTTATTGATAAGGTGACAGGTCAGTGGAAGTTTCCGATGATGTTCAGCCCTTCAAAATCCCTTCAGATAGGCTTAAAAAGCCATTAAATAAGCTTCTTGGTGATGTCGGGTCGAGGTGACCTGATAGTGGCTTGGCAGGTGACCTTCTTAGAGTGGAGAAGGCGGGCATCTAGACTGAGGTCAGGCTCATCCAGTGGCTGAGGAGGCATGGTCCG


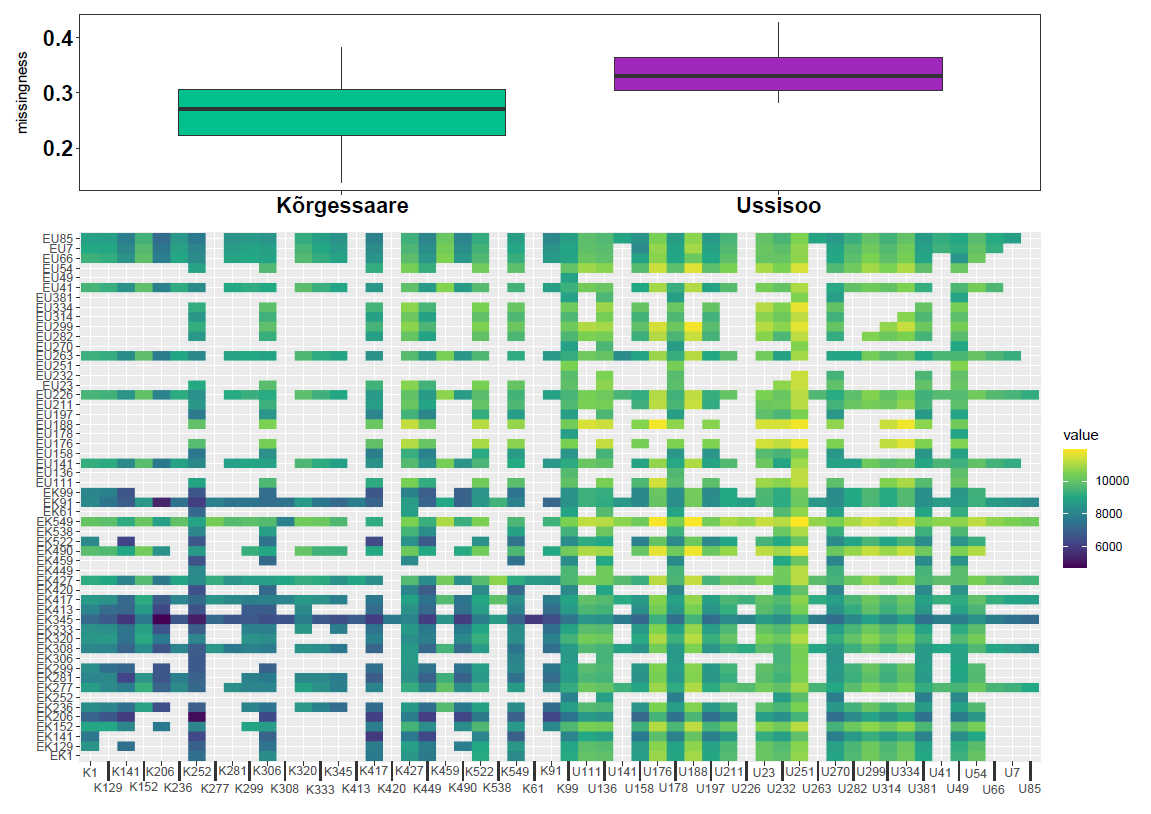


Figure S10. Percentage of missing data per population in the fineRADstructure analysis (boxplots) and heatmap of missingness between pairs of individuals, with lighter colours indicating a higher number of missing data (individual codes below have been abbreviated).

Table S1. Long-term demographic observations of *C. calceolus* populations at Kõrgessaare and Ussisoo (notice that the plot size of the area under consideration is different in the two populations).

|  | **Number of ramets** | |
| --- | --- | --- |
| year | **Ussisoo (plot size: 100 m^2^)** | **Kõrgessaare (plot size: 2 m^2^)** |
| 1978 | 68 | − |
| 1979 | 70 | − |
| 1982 | 98 | − |
| 1983 | 73 | − |
| 1984 | 88 | − |
| 1985 | 146 | 85 |
| 1986 | 120 | 92 |
| 1987 | 137 | 101 |
| 1988 | 173 | 96 |
| 1989 | 162 | 139 |
| 1990 | 111 | 104 |
| 1991 | 165 | 117 |
| 1992 | 228 | 139 |
| 1993 | 199 | 113 |
| 1994 | 204 | 233 |
| 1995 | 181 | 208 |
| 1996 | 167 | 242 |
| 1997 | 149 | 202 |
| 1998 | 193 | 186 |
| 1999 | 179 | 239 |
| 2000 | 137 | 266 |
| 2001 | 199 | 229 |
| 2002 | 168 | 189 |
| 2003 | 180 | 206 |
| 2004 | 190 | − |
| 2005 | 201 | − |
| 2006 | 190 | − |
| 2007 | 204 | − |
| 2008 | 240 | − |
| 2009 | 124 | − |
| 2010 | 224 | − |
| 2011 | 179 | − |
| 2012 | 214 | − |
| 2013 | 161 | − |
| 2014 | 179 | − |
| 2015 | 208 | − |

Table S2. Details of the individuals of *C. calceolus* selected for ddRADseq analysis.

| **IndividualCode** | **Population** | **Ho** | **Code for the corresponding MLL of the SSR data set (see Figure 2)** | **Number of ramets for the corresponding MLL of the SSR data set** | **COMMENTS** |
| --- | --- | --- | --- | --- | --- |
| EU10 | Ussisoo | 0.28 | 251 | 1 | also technical replicate |
| EU49 | Ussisoo | 0.28 | 196 | 16 |  |
| EU178 | Ussisoo | 0.26 | 228 | 5 |  |
| EU251 | Ussisoo | 0.25 | 181 | 2 |  |
| EU136 | Ussisoo | 0.26 | 245 | 10 |  |
| EU270 | Ussisoo | 0.27 | 74 | 12 |  |
| EU381 | Ussisoo | 0.28 | 184 | 20 |  |
| EU232 | Ussisoo | 0.27 | 157 | 5 |  |
| EU23 | Ussisoo | 0.27 | 180 | 11 |  |
| EU197 | Ussisoo | 0.28 | 247 | 6 |  |
| EU158 | Ussisoo | 0.26 | 76 | 35 |  |
| EU334 | Ussisoo | 0.26 | 233 | 4 |  |
| EU314 | Ussisoo | 0.26 | 143 | 9 |  |
| EU176 | Ussisoo | 0.25 | 188 | 19 |  |
| EU111 | Ussisoo | 0.24 | 236 | 13 |  |
| EU367 | Ussisoo | 0.23 | 174 | 13 |  |
| EU188 | Ussisoo | 0.25 | 193 | 4 |  |
| EU299 | Ussisoo | 0.26 | 248 | 2 |  |
| EU78 | Ussisoo | 0.24 | 252 | 19 |  |
| EU282 | Ussisoo | 0.28 | 199 | 12 |  |
| EU421 | Ussisoo | 0.22 | 242 | 26 |  |
| EU43 | Ussisoo | 0.22 | 182 | 1 |  |
| EU211 | Ussisoo | 0.26 | 221 | 29 |  |
| EU54 | Ussisoo | 0.24 | 197 | 13 |  |
| EU66 | Ussisoo | 0.28 | 191 | 4 |  |
| EU41 | Ussisoo | 0.26 | 227 | 9 |  |
| EU7 | Ussisoo | 0.26 | 179 | 9 |  |
| EU141 | Ussisoo | 0.29 | 175 | 19 |  |
| EU263 | Ussisoo | 0.28 | 229 | 1 |  |
| EU85 | Ussisoo | 0.26 | 214 | 17 |  |
| EU226 | Ussisoo | 0.25 | 246 | 3 |  |
| EK252 | Korgessaare | 0.29 | 116 | 1 |  |
| EK449 | Korgessaare | 0.52 | 243 | 18 |  |
| EK61 | Korgessaare | 0.40 | 107 | 8 |  |
| EK306 | Korgessaare | 0.36 | 48 | 11 |  |
| EK336 | Korgessaare | 0.31 | 67 | 6 | putative biological replicate pair EK333-EK336 |
| EK459 | Korgessaare | 0.32 | 72 | 9 |  |
| EK538 | Korgessaare | 0.37 | 73 | 4 |  |
| EK420 | Korgessaare | 0.28 | 149 | 6 |  |
| EK1 | Korgessaare | 0.41 | 166 | 19 |  |
| EK141 | Korgessaare | 0.30 | 207 | 10 |  |
| EK522 | Korgessaare | 0.22 | 145 | 24 |  |
| EK129 | Korgessaare | 0.34 | 101 | 11 |  |
| EK99 | Korgessaare | 0.37 | 114 | 7 |  |
| EK206 | Korgessaare | 0.23 | 139 | 6 | putative biological replicate pair EK206-EK471 |
| EK299 | Korgessaare | 0.41 | 55 | 2 |  |
| EK493 | Korgessaare | 0.30 | 108 | 6 |  |
| EK152 | Korgessaare | 0.34 | 88 | 9 |  |
| EK320 | Korgessaare | 0.53 | 44 | 29 |  |
| EK413 | Korgessaare | 0.38 | 104 | 4 |  |
| EK333 | Korgessaare | 0.38 | 68 | 3 | putative biological replicate pair EK206-EK471 |
| EK471 | Korgessaare | 0.18 | 140 | 21 |  |
| EK490 | Korgessaare | 0.34 | 69 | 10 |  |
| EK236 | Korgessaare | 0.38 | 79 | 12 |  |
| EK281 | Korgessaare | 0.33 | 208 | 30 |  |
| EK277 | Korgessaare | 0.40 | 126 | 22 |  |
| EK308 | Korgessaare | 0.34 | 103 | 40 | putative biological replicate pair EK308-EK549 |
| EK549 | Korgessaare | 0.34 | 103 | 40 | putative biological replicate pair EK308-EK549 |
| EK189 | Korgessaare | 0.18 | 148 | 30 |  |
| EK417 | Korgessaare | 0.35 | 133 | 5 |  |
| EK91 | Korgessaare | 0.22 | 119 | 6 |  |
| EK427 | Korgessaare | 0.24 | 203 | 7 |  |
| EK345 | Korgessaare | 0.11 | 22 | 6 |  |

Ho: Individual observed heterozygosity. In red, samples removed in the “reduced” data set, see Table S3

Table S3 Details of the filtering strategies for the ddRADseq data set in *C. calceolus*. Population codes: USS: Ussisoo, KÕR: Kõrgessaare

| **Code for the filtering strategy^1^** | **Filter type in Stacks *populations*** | **Downstream analyses and rationale** | ***Number of individuals per locus (as output by Stacks* populations*)*** | **#Retained loci** | **#Retained polymorphic loci** | **#Retained variant sites** | ***H*_O_** | ***H*_E_** | **π** | **Private alleles** | **#Loci (and #variant sites) out of HW proportions^2^** | ***F*_IS_** |
| --- | --- | --- | --- | --- | --- | --- | --- | --- | --- | --- | --- | --- |
| r80 | *-r* 0.8  **--*write-single-snp*** | -BayeScan (requires unlinked SNPs)  (all individuals included) | USS: 28.8  KÕR: 28.8 | **32,183** | **27,770** | 27,910  USS: 20,780  KÕR: 22,696 | USS: 0.178  KÕR: 0.169 | USS: 0.186  KÕR: 0.179 | USS: 0.189  KÕR: 0.182 | USS: 8953  KÕR: 1332 | USS: 4114 loci  KÕR: 13,455 loci | USS: 0.045  KÕR: 0.041 |
| reduced | *-r* 0.8  *--min-mac* 3  *--max-obs-het* 0.7  --blacklist (mitochondrial loci and BayeScan outliers) | -Genetic diversity parameters, including private alleles  -Rationale: technical/biological replicates and individuals with a high proportion of missing data are excluded, to avoid underestimation of genetic diversity | USS: 24.4  KÕR: 24.9 | **42,650** | **34,484** | 145,011  USS: 108,106  KÕR: 86,006 | USS: 0.241  KÕR: 0.214 | USS:0.254  KÕR: 0.227 | USS: 0.259  KÕR: 0.232 | USS: 40,268  KÕR: 8734 | USS: 6406 loci (77 sites after FDR correction for multiple comparisons)  KÕR: 11,544 loci  (184 sites after FDR correction for multiple comparisons) | USS: 0.059  KÕR: 0.0497 |
| p2reduced | *-r* 0.8  *--min-mac* 3  *-p* 2  *--max-obs-het* 0.7  --blacklist (mitochondrial loci and BayeScan outliers) | -fineRADstructure  -Rationale: only loci shared by the two populations (p2) are included. Technical replicate and individuals with a high proportion of missing data are excluded, to avoid bias on genetic structure | USS: 24.7  KÕR: 26.1 | **23,804** | **20,366** | 94,921  USS: 94,921  KÕR: 94,921 | USS: 0.239  KÕR: 0.184 | USS: 0.249  KÕR: 0.192 | USS: 0.254  KÕR: 0.195 | USS: 38995  KÕR: 8377 | USS: 5509 loci  KÕR: 7547 loci | USS: 0.046  KÕR: 0.031 |
| forNe | *-r* 0.8  *--min-mac* 3  *--max-obs-het* 0.7  --blacklist (mitochondrial loci and BayeScan outliers)  *--write-random-snp* | -NeEstimator (LD method)  -Rationale: only one SNP at each locus is used, to avoid the influence of physical linkage. As having a larger sample size is more important than having more SNPs (Luikart et al. 2020) we included all samples except the only likely clone and the technical replicate.  From this, we subsampled 40 data sets of 800 SNPs each; see Table 3 in the main manuscript. | USS: 27.8  KÕR: 27.8 | **34,055** | **27,136** | 27,136  USS: 20,398  KÕR: 22,402 | USS: 0.232  KÕR: 0.216 | USS: 0.244  KÕR: 0.232 | USS: 0.249  KÕR: 0.236 | USS: 6803  KÕR: 1361 | USS: 5586 loci  (22 sites after FDR correction for multiple comparisons)  KÕR: 13,437 loci  (88 sites after FDR correction for multiple comparisons) | USS: 0.057  KÕR: 0.054 |

*H*_O_: observed heterozygosity; *H*_E_: expected heterozygosity; π: average nucleotide diversity; *F*_IS_: Inbreeding coefficient.

^1^plastid reads were excluded from all data sets (see Gargiulo et al., 2021);

^2^Loci deviating from the Hardy-Weinberg proportions as output by Stacks populations, without correction for multiple comparisons, unless specified in parentheses (FDR = false discovery rate).

Table S4. Results of the exact tests for heterozygosity excess and deficit on the microsatellite data sets of *Cypripedium calceolus.* Highlighted loci significantly deviate from random mating; significance level (α) was adjusted using Bonferroni correction for multiple testing (α/number of loci = 0.05/11 = 0.0045).

W&C: *F*_IS_ estimates according to Weir & Cockerham (1984); R&H: *F*_IS_ estimates according to Robertson & Hill (1984).

| **MLG clone-corrected (genet) data set** |  |  |  |  |  |  |  |  |  |  |  |  |  |  |
| --- | --- | --- | --- | --- | --- | --- | --- | --- | --- | --- | --- | --- | --- | --- |
| **Ussisoo population** |  |  |  |  |  |  |  |  |  |  |  |  |  |  |
| H1= heterozygote excess |  |  |  |  |  |  |  | H1= heterozygote deficit |  |  |  |  |  |  |
| locus | P-val | S.E. | W&C | R&H | Steps |  |  | locus | P-val | S.E. | W&C | R&H | Steps |  |
| Ccal_7 | 0.6459 | 0.0203 | -0.0538 | -0.0152 | 1777 | switches |  | Ccal_7 | 1 | 0 | -0.0538 | -0.0152 | 1733 | switches |
| Ccal_24 | 0.5637 | 0.0169 | 0.0342 | 0.0048 | 23531 | switches |  | Ccal_24 | 0.431 | 0.0176 | 0.0342 | 0.0048 | 23665 | switches |
| Ccal_25 | 0.0012 | 0.0008 | -0.1465 | -0.0954 | 27295 | switches |  | Ccal_25 | 0.9992 | 0.0004 | -0.1465 | -0.0954 | 27396 | switches |
| Ccal_34 | 0.3103 | 0.0235 | -0.0362 | -0.0211 | 8242 | switches |  | Ccal_34 | 0.6888 | 0.0314 | -0.0362 | -0.0211 | 8118 | switches |
| Ccal_47 | 0.4026 | 0.0143 | 0.0142 | -0.0183 | 16923 | switches |  | Ccal_47 | 0.623 | 0.0134 | 0.0142 | -0.0183 | 17290 | switches |
| C_cal_48 | 0.9981 | 0.0007 | 0.2005 | 0.1596 | 5119 | switches |  | C_cal_48 | 0.0047 | 0.0012 | 0.2005 | 0.1596 | 5186 | switches |
| C_cal_49 | 0.1722 | 0.016 | -0.0509 | -0.0321 | 11060 | switches |  | C_cal_49 | 0.8519 | 0.0133 | -0.0509 | -0.0321 | 11205 | switches |
| C_cal_50 | 0.8183 | - | 0.1297 | 0.0716 | 410 | matrices |  | C_cal_50 | 0.185 | - | 0.1297 | 0.0716 | 410 | matrices |
| C_cal_53 | 1 | 0 | 0.4081 | 0.3035 | 26204 | switches |  | C_cal_53 | 0.0001 | 0.0001 | 0.4081 | 0.3035 | 26622 | switches |
| IK9 | 0.3767 | 0.0164 | 0.0532 | -0.0203 | 32497 | switches |  | IK9 | 0.6212 | 0.0143 | 0.0532 | -0.0203 | 32688 | switches |
| IM30B | 0.1165 | - | -0.0599 | -0.0874 | 100 | matrices |  | IM30B | 0.8864 | - | -0.0599 | -0.0874 | 100 | matrices |
| **Kõrgessaare population** |  |  |  |  |  |  |  |  |  |  |  |  |  |  |
| H1= heterozygote excess |  |  |  |  |  |  |  | H1= heterozygote deficit |  |  |  |  |  |  |
| locus | P-val | S.E. | W&C | R&H | Steps |  |  | locus | P-val | S.E. | W&C | R&H | Steps |  |
| Ccal_7 | 0.0692 | - | -0.1116 | -0.0588 | 263 | matrices |  | Ccal_7 | 0.9483 | - | -0.1116 | -0.0588 | 263 | matrices |
| Ccal_24 | 0.5899 | - | 0.0535 | 0.0045 | 82612 | matrices |  | Ccal_24 | 0.4134 | - | 0.0535 | 0.0045 | 82612 | matrices |
| Ccal_25 | 0.779 | 0.0263 | 0.025 | 0.0145 | 9715 | switches |  | Ccal_25 | 0.2285 | 0.0393 | 0.025 | 0.0145 | 9713 | switches |
| Ccal_34 | 0.5871 | - | 0.0596 | 0.0061 | 90729 | matrices |  | Ccal_34 | 0.4129 | - | 0.0596 | 0.0061 | 90729 | matrices |
| Ccal_47 | 0.9259 | - | 0.1258 | 0.0527 | 250 | matrices |  | Ccal_47 | 0.0746 | - | 0.1258 | 0.0527 | 250 | matrices |
| C_cal_48 | 0.9942 | 0.0017 | 0.1887 | 0.1643 | 613 | switches (low!) | | C_cal_48 | 0.0088 | 0.003 | 0.1887 | 0.1643 | 570 | switches (low!) |
| C_cal_49 | 0.4578 | - | -0.0014 | -0.0078 | 148294 | matrices |  | C_cal_49 | 0.5552 | - | -0.0014 | -0.0078 | 148294 | matrices |
| C_cal_50 | 0.7329 | - | 0.0342 | 0.0343 | 82 | matrices |  | C_cal_50 | 0.3726 | - | 0.0342 | 0.0343 | 82 | matrices |
| C_cal_53 | 0.999 | - | 0.2143 | 0.2004 | 18108 | matrices |  | C_cal_53 | 0.0011 | - | 0.2143 | 0.2004 | 18108 | matrices |
| IK9 | 0.9999 | 0.0001 | -0.0005 | 0.1847 | 14496 | switches |  | IK9 | 0.0005 | 0.0004 | -0.0005 | 0.1847 | 14632 | switches |
| IM30B | 0 | - | -0.016 | -0.0161 | 4 | matrices |  | IM30B | 1 | - | -0.016 | -0.0161 | 4 | matrices |
|  |  |  |  |  |  |  |  |  |  |  |  |  |  |  |
| **MLL clone-corrected datasets** |  |  |  |  |  |  |  |  |  |  |  |  |  |  |
| **Ussisoo population** |  |  |  |  |  |  |  |  |  |  |  |  |  |  |
| H1= heterozygote excess | P-val | S.E. | W&C | R&H | Steps |  |  | H1= heterozygote deficit | P-val | S.E. | W&C | R&H | Steps |  |
| Ccal_7 | 0.7283 | 0.0194 | -0.0471 | -0.0122 | 1435 | switches |  | Ccal_7 | 1 | 0 | -0.0471 | -0.0122 | 1230 | switches |
| Ccal_24 | 0.5397 | 0.0146 | 0.0273 | -0.0024 | 21847 | switches |  | Ccal_24 | 0.4292 | 0.0154 | 0.0273 | -0.0024 | 21864 | switches |
| Ccal_25 | 0.004 | 0.0013 | -0.1464 | -0.0922 | 23980 | switches |  | Ccal_25 | 0.9959 | 0.0012 | -0.1464 | -0.0922 | 24216 | switches |
| Ccal_34 | 0.4067 | 0.0365 | -0.0059 | -0.0133 | 7640 | switches |  | Ccal_34 | 0.6018 | 0.0279 | -0.0059 | -0.0133 | 7920 | switches |
| Ccal_47 | 0.4806 | 0.014 | 0.0355 | -0.0088 | 15705 | switches |  | Ccal_47 | 0.5265 | 0.0226 | 0.0355 | -0.0088 | 15459 | switches |
| C_cal_48 | 0.9988 | 0.0006 | 0.2117 | 0.1625 | 5201 | switches |  | C_cal_48 | 0.0054 | 0.0019 | 0.2117 | 0.1625 | 5303 | switches |
| C_cal_49 | 0.3207 | 0.0202 | -0.0233 | -0.0197 | 10797 | switches |  | C_cal_49 | 0.6611 | 0.031 | -0.0233 | -0.0197 | 10726 | switches |
| C_cal_50 | 0.8768 | - | 0.1773 | 0.1015 | 284 | matrices |  | C_cal_50 | 0.1269 | - | 0.1773 | 0.1015 | 284 | matrices |
| C_cal_53 | 1 | 0 | 0.4612 | 0.3445 | 25588 | switches |  | C_cal_53 | 0 | 0 | 0.4612 | 0.3445 | 25729 | switches |
| IK9 | 0.439 | 0.0119 | 0.062 | -0.0157 | 31597 | switches |  | IK9 | 0.5545 | 0.0149 | 0.062 | -0.0157 | 31562 | switches |
| IM30B | 0.0849 | - | -0.0859 | -0.101 | 100 | matrices |  | IM30B | 0.9181 | - | -0.0859 | -0.101 | 100 | matrices |
|  |  |  |  |  |  |  |  |  |  |  |  |  |  |  |
| Kõrgessaare population |  |  |  |  |  |  |  |  |  |  |  |  |  |  |
| H1= heterozygote excess | P-val | S.E. | W&C | R&H | Steps |  |  | H1= heterozygote deficit | P-val | S.E. | W&C | R&H | Steps |  |
| Ccal_7 | 0.3594 | - | -0.0349 | -0.0201 | 222 | matrices |  | Ccal_7 | 0.699 | - | -0.0349 | -0.0201 | 222 | matrices |
| Ccal_24 | 0.8184 | - | 0.123 | 0.0329 | 46570 | matrices |  | Ccal_24 | 0.1834 | - | 0.123 | 0.0329 | 46570 | matrices |
| Ccal_25 | 0.586 | 0.0449 | 0.0048 | 0.007 | 9843 | switches |  | Ccal_25 | 0.3051 | 0.04 | 0.0048 | 0.007 | 9713 | switches |
| Ccal_34 | 0.5445 | - | 0.0483 | 0.0013 | 48734 | matrices |  | Ccal_34 | 0.4597 | - | 0.0483 | 0.0013 | 48734 | matrices |
| Ccal_47 | 0.8681 | - | 0.1128 | 0.0434 | 202 | matrices |  | Ccal_47 | 0.1328 | - | 0.1128 | 0.0434 | 202 | matrices |
| C_cal_48 | 0.9949 | 0.0017 | 0.2107 | 0.1646 | 723 | switches (low!) | | C_cal_48 | 0.0179 | 0.003 | 0.2107 | 0.1646 | 579 | switches (low!) |
| C_cal_49 | 0.4173 | - | -0.0033 | -0.0123 | 54794 | matrices |  | C_cal_49 | 0.5997 | - | -0.0033 | -0.0123 | 54794 | matrices |
| C_cal_50 | 0.6452 | - | 0.0164 | 0.0164 | 65 | matrices |  | C_cal_50 | 0.4831 | - | 0.0164 | 0.0164 | 65 | matrices |
| C_cal_53 | 0.9992 | - | 0.2614 | 0.2501 | 10455 | matrices |  | C_cal_53 | 0.0008 | - | 0.2614 | 0.2501 | 10455 | matrices |
| IK9 | 0.9993 | 0.0004 | 0.0051 | 0.1895 | 14526 | switches |  | IK9 | 0.0008 | 0.0005 | 0.0051 | 0.1895 | 14268 | switches |
| IM30B | 0.934 | - | -0.0196 | -0.0197 | 4 | matrices |  | IM30B | 1 | - | -0.0196 | -0.0197 | 4 | matrices |
|  |  |  |  |  |  |  |  |  |  |  |  |  |  |  |
| **Raw data set (ramet data set)** |  |  |  |  |  |  |  |  |  |  |  |  |  |  |
| **Ussisoo population** |  |  |  |  |  |  |  |  |  |  |  |  |  |  |
| H1= heterozygote excess | P-val | S.E. | W&C | R&H | Steps |  |  | H1= heterozygote deficit | P-val | S.E. | W&C | R&H | Steps |  |
| Ccal_7 | 0.0009 | 0.0005 | -0.0757 | -0.0286 | 19006 | switches |  | Ccal_7 | 1.0000 | 0.0000 | -0.0757 | -0.0286 | 18887 | switches |
| Ccal_24 | 0.7216 | 0.0212 | 0.0356 | 0.0087 | 51154 | switches |  | Ccal_24 | 0.3652 | 0.0356 | 0.0356 | 0.0087 | 51205 | switches |
| Ccal_25 | 0.0000 | 0.0000 | -0.1284 | -0.0680 | 47795 | switches |  | Ccal_25 | 1.0000 | 0.0000 | -0.1284 | -0.0680 | 47544 | switches |
| Ccal_34 | 0.0169 | 0.0093 | -0.0469 | -0.0259 | 27736 | switches |  | Ccal_34 | 0.9865 | 0.0071 | -0.0469 | -0.0259 | 27940 | switches |
| Ccal_47 | 0.1314 | 0.0212 | 0.0352 | -0.0220 | 29108 | switches |  | Ccal_47 | 0.8875 | 0.0199 | 0.0352 | -0.0220 | 29152 | switches |
| C_cal_48 | 0.9905 | 0.0013 | 0.1002 | 0.0624 | 13156 | switches |  | C_cal_48 | 0.0044 | 0.0011 | 0.1002 | 0.0624 | 12992 | switches |
| C_cal_49 | 0.0000 | 0.0000 | -0.0946 | -0.0468 | 27449 | switches |  | C_cal_49 | 1.0000 | 0.0000 | -0.0946 | -0.0468 | 27399 | switches |
| C_cal_50 | 1.0000 | - | 0.2892 | 0.1659 | 104958 | matrices |  | C_cal_50 | 0.0000 | - | 0.2892 | 0.1659 | 104958 | matrices |
| C_cal_53 | 1.0000 | 0.0000 | 0.4920 | 0.2908 | 55418 | switches |  | C_cal_53 | 0.0000 | 0.0000 | 0.4920 | 0.2908 | 55281 | switches |
| IK9 | 0.0001 | 0.0001 | -0.0123 | -0.0601 | 68778 | switches |  | IK9 | 0.9999 | 0.0000 | -0.0123 | -0.0601 | 68731 | switches |
| IM30B | 0.0000 | - | -0.1349 | -0.1303 | 40140 | matrices |  | IM30B | 1.0000 | - | -0.1349 | -0.1303 | 40140 | matrices |
|  |  |  |  |  |  |  |  |  |  |  |  |  |  |  |
| **Kõrgessaare population** |  |  |  |  |  |  |  |  |  |  |  |  |  |  |
| H1= heterozygote excess | P-val | S.E. | W&C | R&H | Steps |  |  | H1= heterozygote deficit | P-val | S.E. | W&C | R&H | Steps |  |
| Ccal_7 | 0.0000 | - | -0.1572 | -0.0809 | 3190 | matrices |  | Ccal_7 | 1.0000 | - | -0.1572 | -0.0809 | 3190 | matrices |
| Ccal_24 | 0.0318 | - | -0.0418 | -0.0340 | 8713007 | matrices |  | Ccal_24 | 0.9682 | - | -0.0418 | -0.0340 | 8713007 | matrices |
| Ccal_25 | 0.6779 | 0.0594 | -0.0328 | 0.0049 | 10345 | switches |  | Ccal_25 | 0.2726 | 0.0543 | -0.0328 | 0.0049 | 10228 | switches |
| Ccal_34 | 0.8845 | - | 0.1030 | 0.0225 | 5160694 | matrices |  | Ccal_34 | 0.1155 | - | 0.1030 | 0.0225 | 5160694 | matrices |
| Ccal_47 | 0.9976 | - | 0.1672 | 0.0791 | 604 | matrices |  | Ccal_47 | 0.0024 | - | 0.1672 | 0.0791 | 604 | matrices |
| C_cal_48 | 0.9998 | 0.0001 | 0.1966 | 0.1660 | 153 | switches (low!) |  | C_cal_48 | 0.0012 | 0.0009 | 0.1966 | 0.1660 | 156 | switches (low!) |
| C_cal_49 | 0.9698 | - | 0.0712 | 0.0381 | 10085619 | matrices |  | C_cal_49 | 0.0302 | - | 0.0712 | 0.0381 | 10085619 | matrices |
| C_cal_50 | 0.0029 | - | -0.1129 | -0.1129 | 306 | matrices |  | C_cal_50 | 0.9971 | - | -0.1129 | -0.1129 | 306 | matrices |
| C_cal_53 | 0.9999 | - | 0.1410 | 0.1780 | 52512 | matrices |  | C_cal_53 | 0.0001 | - | 0.1410 | 0.1780 | 52512 | matrices |
| IK9 | 0.9990 | 0.0005 | -0.0492 | 0.1173 | 18545 | switches |  | IK9 | 0.0001 | 0.0001 | -0.0492 | 0.1173 | 17919 | switches |
| IM30B | 0.9834 | - | -0.0048 | -0.0048 | 4 | matrices |  | IM30B | 0.0166 | - | -0.0048 | -0.0048 | 4 | matrices |

Table S5. Summary of private alleles by population of *C. calceolus*.

| **Population** | **Locus** | **Allele** | **Freq** | **Info from the data set in Gargiulo et al. (2019)** |
| --- | --- | --- | --- | --- |
| Ussisoo | Ccal_7 | 186 | 0.008 | widespread in Eurasia |
| Ussisoo | Ccal_7 | 192 | 0.023 | widespread in Eurasia |
| Ussisoo | Ccal_24 | 93 | 0.008 | widespread in Eurasia |
| Ussisoo | Ccal_24 | 102 | 0.045 | widespread in Eurasia |
| Ussisoo | Ccal_25 | 137 | 0.068 | widespread in Europe |
| Ussisoo | Ccal_25 | 140 | 0.083 | widespread in Eurasia |
| Ussisoo | Ccal_25 | 144 | 0.159 | widespread in Eurasia |
| Ussisoo | Ccal_25 | 147 | 0.068 | widespread in Eurasia |
| Ussisoo | Ccal_25 | 151 | 0.227 | widespread in Eurasia |
| Ussisoo | Ccal_34 | 108 | 0.356 | UK, Finland, Italy |
| Ussisoo | Ccal_34 | 114 | 0.045 | widespread in Eurasia |
| Ussisoo | Ccal_34 | 116 | 0.015 | widespread in Eurasia |
| Ussisoo | Ccal_34 | 122 | 0.023 | UK, Russian Far East and Italy |
| Ussisoo | Ccal_34 | 132 | 0.008 | Estonia, Finland e Russia |
| Ussisoo | Ccal_47 | 117 | 0.008 | Italy, Romania, France, UK |
| Ussisoo | Ccal_47 | 129 | 0.038 | Northern Europe, Russia and Romania |
| Ussisoo | C_cal_49 | 97 | 0.008 | widespread in Eurasia |
| Ussisoo | C_cal_49 | 127 | 0.008 | widespread in Eurasia |
| Ussisoo | C_cal_49 | 130 | 0.076 | various parts of Europe |
| Ussisoo | C_cal_49 | 133 | 0.008 | widespread in Eurasia |
| Ussisoo | C_cal_50 | 103 | 0.053 | Large-scale data not available |
| Ussisoo | C_cal_53 | 149 | 0.125 | widespread in Eurasia |
| Ussisoo | C_cal_53 | 168 | 0.023 | widespread in Eurasia |
| Ussisoo | C_cal_53 | 171 | 0.078 | widespread in Eurasia |
| Ussisoo | IK9 | 174 | 0.038 | widespread in Eurasia |
| Ussisoo | IM30B | 230 | 0.030 | widespread in Eurasia |
| Kõrgessaare | Ccal_25 | 127 | 0.003 | NEW (not found before) |
| Kõrgessaare | Ccal_25 | 157 | 0.487 | widespread in Eurasia |
| Kõrgessaare | C_cal_53 | 145 | 0.014 | Estonia, UK, Poland, Italy |
| Kõrgessaare | IK9 | 177 | 0.008 | widespread in Europe |
